# FeatureForest: the power of foundation models, the usability of random forests

**DOI:** 10.1038/s44303-025-00089-9

**Published:** 2025-07-08

**Authors:** Mehdi Seifi, Damian Dalle Nogare, Juan Manuel Battagliotti, Vera Galinova, Ananya Kedige Rao, Pierre-Henri Jouneau, Anwai Archit, AI4Life Horizon Europe Programme Consortium, Fynn Beuttenmueller, Dorothea Dörr, Mariana G. Ferreira, Caterina Fuster-Barceló, Vera Galinova, Carlos García-López-de-Haro, Estibaliz Gómez-de-Mariscal, Matthew Hartley, Ricardo Henriques, Iván Hidalgo-Cenalmor, Florian Jug, Anna Kreshuk, Emma Lundberg, Nils Mechtel, Arrate Muñoz-Barrutia, Wei Ouyang, Constantin Pape, Craig T. Russell, Mehdi Seifi, Beatriz Serrano-Solano, Tomaz Vieira, Teresa Zulueta-Coarasa, Joran Deschamps, Constantin Pape, Johan Decelle, Florian Jug, Joran Deschamps

**Affiliations:** 1 Computational Biology Research Center, Human Technopole, Milan, Italy; 2 Bioimage Analysis Unit, National Facility for Data Handling and Analysis, Human Technopole, Milan, Italy; 3 Cell and Plant Physiology Laboratory, CNRS, CEA, INRAE, IRIG, Université Grenoble Alpes, Grenoble, France; 4 University Grenoble Alpes, CEA, IRIG-MEM, 38054 Grenoble, France; 5 Institute of Computer Science, University of Göttingen, Göttingen, Germany; 6 Cluster of Excellence “Multiscale Bioimaging: from Molecular Machines to Networks of Excitable Cells”, University of Göttingen, Göttingen, Germany; 7 Cell Biology and Biophysics Unit, European Molecular Biology Laboratory, Heidelberg, Germany; 8 Euro-BioImaging ERIC Statutory Seat, Turku, Finland; 9 AI-driven Optical Biology Laboratory, Instituto de Tecnologia Química e Biológica António Xavier, Universidade Nova de Lisboa, Oeiras, Portugal; 10 Optical Cell Biology Laboratory, Instituto Gulbenkian de Ciência, Oeiras, Portugal; 11 Optical Cell Biology Laboratory, Gulbenkian Institute for Molecular Medicine, Oeiras, Portugal; 12 Bioengineering Department, Universidad Carlos III de Madrid, Leganes, Spain; 13 Biological Image Analysis Unit, Institut Pasteur, Paris, France; 14 European Molecular Biology Laboratory, European Bioinformatics Institute, Hinxton, UK; 15 UCL Laboratory for Molecular Cell Biology, University College London, London, UK; 16 Stanford University, Stanford, CA USA; 17 SciLifeLab, KTH Royal Institute of Technology, Stockholm, Sweden; 18 Euro-Bioimaging ERIC Bio-Hub, European Molecular Biology Laboratory, Heidelberg, Germany

**Keywords:** Computational biology and bioinformatics, Image processing

## Abstract

Analysis of biological images relies heavily on segmenting the biological objects of interest in the image before performing quantitative analysis. Deep learning (DL) is ubiquitous in such segmentation tasks, but can be cumbersome to apply, as it often requires a large amount of manual labeling to produce ground-truth data, and expert knowledge to train the models. More recently, large foundation models, such as SAM, have shown promising results on scientific images. They, however, require manual prompting for each object or tedious post-processing to selectively segment these objects. Here, we present FeatureForest, a method that leverages the feature embeddings of large foundation models to train a random forest classifier, thereby providing users with a rapid way of semantically segmenting complex images using only a few labeling strokes. We demonstrate the improvement in performance over a variety of datasets and provide an open-source implementation in napari that can be extended to new models.

## Introduction

Segmentation is a ubiquitous task in microscopy image analysis, as it enables downstream processing and quantification of objects of interest. Researchers have at their disposal a wide array of algorithms, among which machine learning approaches have long been the methods of choice. In particular, random forest pixel classification is a well-established algorithm, at the heart of several popular software tools for bioimage analysis^1–4^. This approach uses common image filters to extract a feature vector representation of hand-labeled pixels in order to train decision trees to best match the given input labels. Because the image filters can be 2D or 3D, random forest pixel classifiers can natively perform 3D segmentation. Moreover, they are compatible with multiclass pixel classification. These algorithms owe their popularity to the simple iterative process by which users draw small scribbles to assign a class to a subset of pixels, rapidly train a random forest, and predict results over many images. This swift training procedure allows the correction of mistakes by adding new labels to the training set and training anew. While random forest pixel classification algorithms have a wide application range covering all types of images and modalities, they are limited in their predictive power, and easily confuse different object types that have similar textures^3^.

In recent years, deep learning has emerged as the most powerful approach for image segmentation. Such approaches are most often trained in a supervised fashion, that is to say, with a large dataset of manually segmented images as reference^5,6^. The likes of StarDist^7^ or CellPose^8^ are go-to tools for image analysts wanting to perform image segmentation. Once trained, these methods often outperform random forest pixel classification^9,10^ and are compatible with 3D segmentation. Furthermore, CellPose2^11^ introduced user-friendly fine-tuning of models by providing a user interface to correct errors and retrain the selected model, similar to the way random forest classifiers are used. Base CellPose models were trained on datasets consisting of various imaging modalities and diverse samples, and are capable of segmenting objects of similar size in a wide range of images. It does not, however, segment multiple classes, and can struggle to effectively segment objects with various shapes and sizes simultaneously.

With more compute power and more data being available, much larger networks are now being trained with astounding results. For instance, Segment Anything Model (SAM)^12^, is capable of accurately segmenting biological objects in 2D in both electron and light microscopy images, all the while being trained on a dataset overwhelmingly composed of natural (i.e. every-day scenes) images. To push the boundary of its capabilities, fine-tuning this model with scientific images is being explored^13–17^.

SAM does not natively segment whole images, but rather expects user annotations - also called prompts - in the form of bounding boxes or points as inputs, and returns segmented instances of the annotated objects. While this is a powerful way to enable interactivity, scientific segmentation pipelines preferentially require automated processing of large datasets. SAM ships with an auto-segmentation method based on automatically generating prompts as a grid of points over the image. Unfortunately, such a feature is not guided and will result, in most cases, in missing objects of interest and other types of object being segmented as well. Without an accurate and automated way of producing the prompts, SAM applications in bioimage analysis are limited to direct and time-consuming user interactions for each object in the dataset.

Another fruitful research avenue is the use of rich latent spaces as basis for segmentation. Rather than segmenting pixels directly, other approaches, such as MAESTER^18^ or DINOv2^19,20^, train a large network on a different task (e.g., reconstructing masked areas of the image) in order to produce rich feature embedding of the image. These features can then be used to cluster the pixels based on their proximity in this latent space, and identify object classes with these clusters. While enticing, cluster-based features are often limited by the lack of knowledge of how many classes are expected in a given image, and whether these classes cluster meaningfully in the feature space. Moreover, the application of such approaches are so far limited to deep-learning experts due to the complexity of the training process, and success in segmenting scientific images different from those in the training set is not ensured.

Here, we present FeatureForest, a method that combines the power of large deep-learning models with the simplicity and user-guidance provided by random forest classification algorithms. With FeatureForest, manual labeling can be a matter of minutes, and user-guidance allows segmenting complex objects throughout entire datasets without requiring re-training large deep learning networks. We showcase how FeatureForest fills a gap in the segmentation of large electron microscopy datasets, enabling researchers to segment challenging images. More specifically, FeatureForest uses large foundation models to extract feature vectors corresponding to user-labeled pixels in order to train a random forest algorithm. In this manuscript, we demonstrate the usefulness of FeatureForest over various scientific datasets for which no straightforward or user-friendly algorithm exists, and the improvements it yields over classical random forest classification. We provide an implementation of FeatureForest in an open napari^21^ plugin, as well as example scripts and notebooks to perform prediction outside napari (e.g., on computer clusters).

## Results

### FeatureForest in a nutshell

**Fig. 1:**
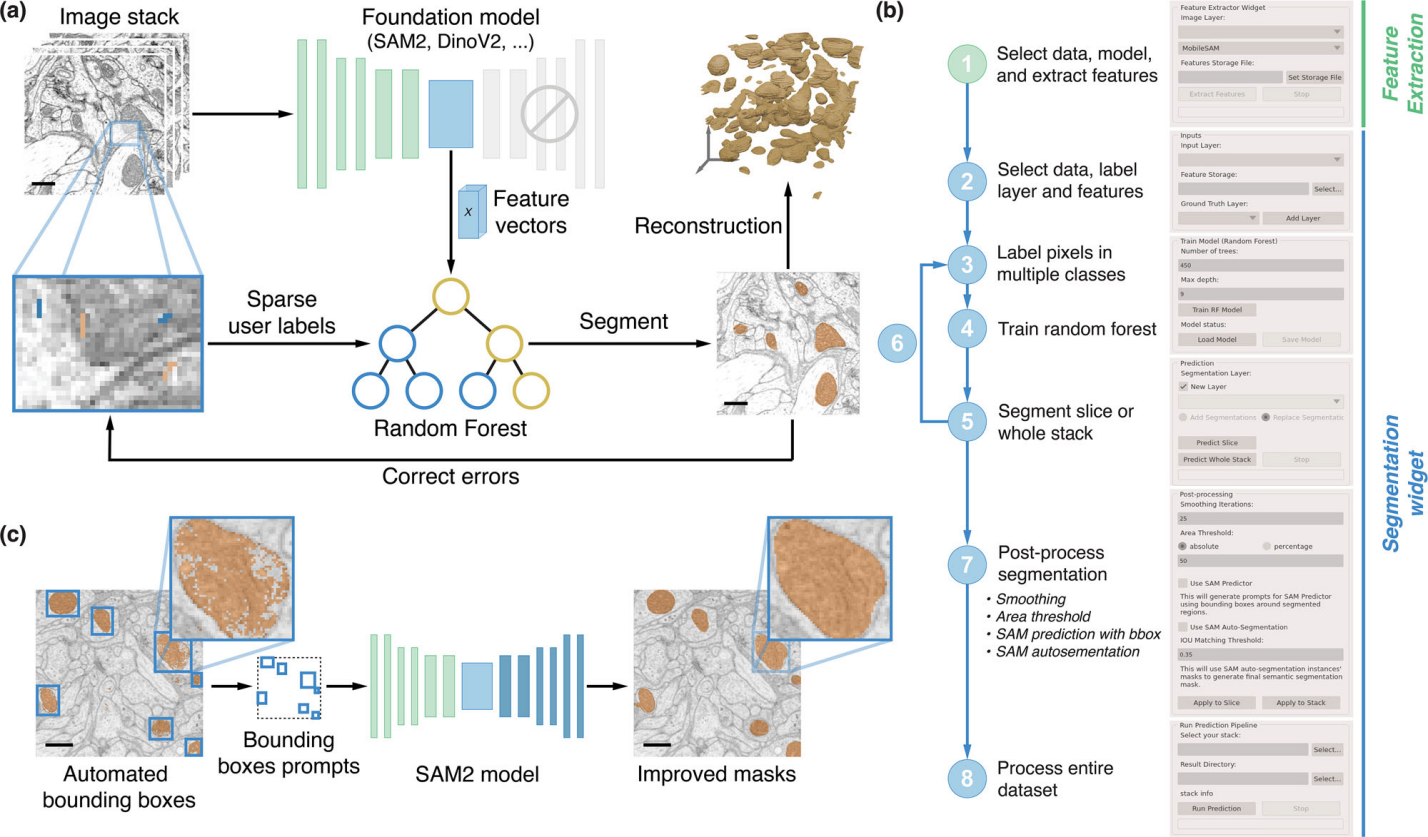
FeatureForest principle. **a** A subset of the data is used to train the random forest model. First, feature vectors from a large deep-learning model corresponding to all pixels in the data are extracted. Users provide both pixel labels and feature vectors to a random forest classifier in order to train the random forest model. Once trained, the classifier can segment slices in the data. An iterative labeling, training, and segmenting process is followed to improve the classifier until satisfaction. **b** Overview of the napari widgets and the various steps followed by users. **c** Post-processing with SAM2 is performed by generating bounding boxes around the connected components in the segmentation, and using them as prompts for SAM2. This results in multiple masks that are merged into a semantic segmentation mask.

FeatureForest replaces the classical filters of a random forest classifier with large deep-learning models (see Fig. 1a), and extracts the feature vectors used during random forest training from the embeddings that are computed within those networks. The overall iterative training process remains otherwise similar, with users requiring a few iterations of labeling and training before obtaining desired results. FeatureForest currently includes several foundation models: MobileSAM^22^, SAM2^23^ and DINOv2^19^ (see Methods for a description of the feature vectors extraction process). Users can extend this list and adapt the model of their choice for use in FeatureForest (see Methods).

In Fig. 1b, we describe the FeatureForest pipeline as available to users via the napari plugin we provide. In a first step, using the *Feature Extraction* widget, users extract the feature vectors corresponding to all pixels in a set of images loaded in napari from the model of their choice. The feature vectors corresponding to individual pixels are stored in an HDF5 file to allow random access during the later stages. The feature vectors are large (from 320 to 1536 features per pixel, depending on the model) and their extraction is slow. Therefore, storing them once for the whole training dataset enables faster iterations when training the random forest.

Then, the *Segmentation Widget* is used to train iteratively a random forest on the data subset, as well as perform the final segmentation. First, users select a napari layer containing their data, point to the feature vector file that was exported using the *Feature Extraction* widget, and select their labeling layer. Next, using the napari’s built-in labeling tools, they label a small representative set of pixels before training a random forest on these labeled pixels. Once the training is done, users can segment the currently selected slice or a full stack. The results can be improved by iteratively adding new labeled pixels where the trained classifier performed poorly. The training process allows rapid iteration between labeling, training, and prediction. At any point, users can save the trained random forest classifier. After a few iterations, users can predict on a dataset saved on the disk, e.g., a much larger stack.

Furthermore, FeatureForest includes post-processing (see Methods), such as smoothing steps and filtering connected components based on size. Additional post-processing tools leverage SAM2 in two different ways: (i) by generating bounding boxes around instances obtained from performing watershed on the output of FeatureForest and using them as prompts for SAM2 (see Fig. 1c), or (ii) by using the SAM2 auto-segmentation feature in which a grid of points over the image is passed to the model as prompts, and the final masks are selected by thresholding the intersection over union (IoU) between instances obtained from SAM2 and watershed-processed FeatureForest results. Using SAM2 in the post-processing step typically results in object segmentations with smoother boundaries (see Fig. 1c insets).

### FeatureForest on various microscopy modalities

We applied FeatureForest to various datasets from three different imaging modalities: focused ion beam scanning electron microscopy (FIB-SEM), label-free microscopy, and H&E staining. For each dataset, we trained a classical random forest classifier using Labkit^3^ and FeatureForest on the same training images. In this section, we used SAM2 as the feature-generating model in FeatureForest, and applied the SAM2 bounding-box post-processing available within FeatureForest (see Methods). In order to quantify the segmentation performance, we computed precision, recall, Dice score, boundary F1^24^, and Hausdorff distance^25^ metrics between the resulting segmentation and the ground-truth provided in the public datasets.

**Fig. 2:**
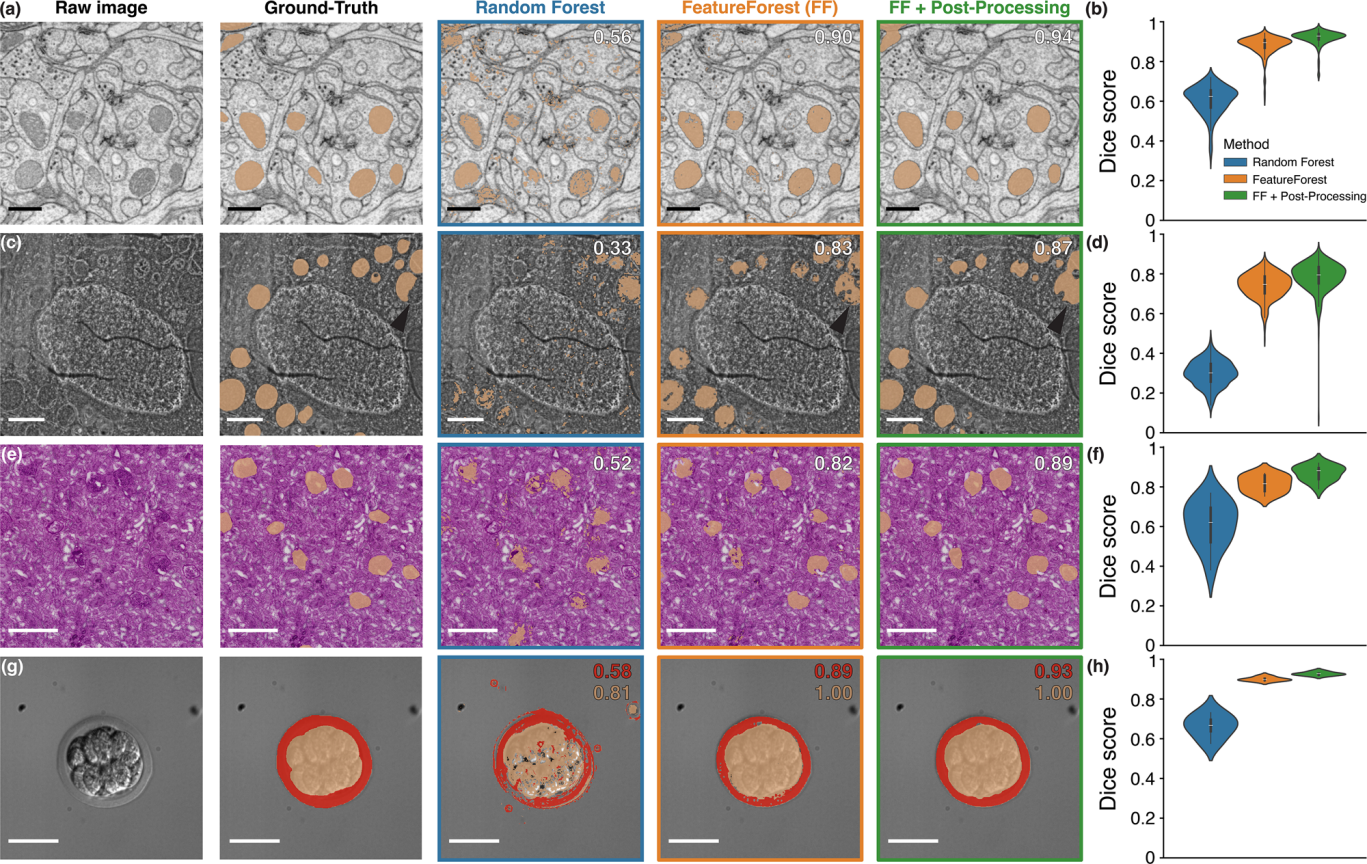
FeatureForest outperforms classical random forest classifiers on complex datasets. **a** FIB-SEM image of a fly brain, overlaid with mitochondria ground-truth mask, and with corresponding segmentation obtained with a random forest classifier, FeatureForest, and after post-processing the results from FeatureForest, from left to right respectively. Dice score with respect to the ground-truth for the specific slice and algorithm is indicated in the top right corner. Scale bar 500 nm. **b** Dice score distribution over the whole dataset (256 slices) presented in (**a**) for the random forest classifier (blue), FeatureForest (orange), and post-processed FeatureForest (green). **c** FIB-SEM image of a human breast cancer spheroid, overlaid with mitochondria ground-truth mask, and with results from a random forest classifier, FeatureForest, and FeatureForest with post-processing, as in (**a**). The arrows indicate incomplete segmentation of a mitochondria instance in the ground-truth that is correctly segmented by FeatureForest. Scale bar 1 μm. **d** Distribution of the Dice score corresponding to (**c**) over the whole dataset (500 slices). **e** H&E staining of a human kidney tissue slice, overlaid with glomerulus ground-truth, and with results from a random forest classifier, FeatureForest, and FeatureForest with post-processing, as in (**a**). Scale bar 500 μm. **f** Distribution of the Dice score corresponding to (**e**) over the whole stack (12 tiles). **g** Label-free brightfield image of a mouse embryo, overlaid with cells (orange) and extraembryonic membrane (red) ground-truth masks, and with results from a random forest classifier, FeatureForest, and FeatureForest with post-processing, as in (**a**). Dice score is indicated for each class. Scale bar 50 μm. **h** Distribution of the Dice score corresponding to the membrane class (red) in (**g**) over the whole stack (5 slices). FeatureForest was trained using *SAM2_Large* for all panels.

FIB-SEM data typically has high contrast and dense structures, while being too large to manually label and too complex to segment using random forest pixel classifiers. Figure 2a shows a single slice of a fly brain imaged by FIB-SEM as well as the mitochondria ground-truth masks, and the segmentations obtained with Labkit and FeatureForest. The mitochondria appear as dark and round objects of varying intensity. While the random forest classifier is able to classify most pixels from inside the mitochondria, it also creates a high number of false positives and misses their outer membrane. In contrast to Labkit, FeatureForest produces a segmentation with high coverage of the mitochondria and few false positive pixels, which is quantitatively confirmed by a Dice score of 0.56 for the random forest and 0.90 for FeatureForest. Post-processing the segmentation from FeatureForest using the bounding boxes generation and SAM2 (see Fig. 1c) yields smoother segmentation masks and a further improved Dice score of 0.94. Similar results are obtained throughout the dataset (see various slices in Supplementary Fig. 1), and computing the Dice score over the entire dataset shows that FeatureForest performs much better than the classical random forest (see Fig. 2b), with mean and standard deviations of 0.88 ± 0.05 (FeatureForest), 0.92 ± 0.04 (FeatureForest + post-processing), and 0.61 ± 0.07 (random forest). In addition to the higher mean Dice score, FeatureForest also results in lower variability and less sensitivity to varying image quality. These results are confirmed across various other metrics (see Table 1 and Supplementary Fig. 2 for the distributions), such as precision and recall, which are used to compute the Dice score but are sensitive to different components of the confusion matrix, as well as boundary-based metrics: boundary F1 and Hausdorff distance.

**Table 1:** Metrics score comparing a random forest classifier, FeatureForest, and FeatureForest with Post-Processing

Dataset	Method	Dice score *↑*	Precision *↑*	Recall *↑*	Boundary F1 *↑*	Hausdorff dist. *↓*
Fly brain	Random Forest	0.61 ± 0.07	0.60 ± 0.09	0.62 ± 0.07	0.38 ± 0.07	11.54 ± 4.72
	FeatureForest (FF)	0.88 ± 0.05	0.91 ± 0.05	0.86 ± 0.06	0.70 ± 0.06	2.82 ± 2.04
	FF + Post-Processing	0.92 ± 0.04	0.97 ± 0.03	0.87 ± 0.05	0.92 ± 0.05	1.49 ± 1.67
Spheroid	Random Forest	0.30 ± 0.06	0.26 ± 0.09	0.38 ± 0.07	0.26 ± 0.08	73.63 ± 26.89
	FeatureForest (FF)	0.74 ± 0.06	0.76 ± 0.09	0.73 ± 0.06	0.71 ± 0.07	10.80 ± 7.74
	FF + Post-Processing	0.78 ± 0.07	0.78 ± 0.10	0.80 ± 0.06	0.79 ± 0.07	9.14 ± 8.27
Kidney	Random Forest	0.61 ± 0.11	0.60 ± 0.13	0.63 ± 0.11	0.50 ± 0.08	21.28 ± 17.41
	FeatureForest (FF)	0.81 ± 0.04	0.79 ± 0.07	0.85 ± 0.05	0.63 ± 0.07	10.53 ± 6.83
	FF + Post-Processing	0.87 ± 0.04	0.86 ± 0.07	0.89 ± 0.05	0.87 ± 0.06	7.16 ± 4.86
Embryo (membrane)	Random Forest	0.66 ± 0.06	0.59 ± 0.09	0.75 ± 0.03	0.48 ± 0.02	39.20 ± 22.63
	FeatureForest (FF)	0.90 ± 0.01	1.00 ± 0.00	0.82 ± 0.02	0.88 ± 0.05	0.65 ± 0.02
	FF + Post-Processing	0.93 ± 0.01	1.00 ± 0.00	0.86 ± 0.02	0.96 ± 0.03	0.50 ± 0.02

The datasets are the same as those shown in Fig. 2: Fly brain (panel (a), *N* = 256), Spheroid (panel (c), *N* = 512), Kidney (panel (e), *N* = 12), and Embryo (panel (g), *N* = 5). The measurements are shown as mean ± standard deviation over the entire dataset. The best performing method for each metric is underlined, where for Dice, precision, recall, and boundary F1 larger values are better (*↑*), while for the Hausdorff distance, smaller is better (*↓*). FeatureForest was trained using *SAM2_Large*.

The example dataset of Fig. 2a is a relatively easy segmentation challenge as the mitochondrial texture is sufficiently different from the rest of the image to be well captured by classical image filters. Classical image analysis could further improve the segmentation obtained with the random forest classifier, for instance, by filtering connected components by size and applying smoothing or morphological operations. In Fig. 2c, we use another FIB-SEM dataset (human breast cancer spheroid) in which the mitochondria have similar texture to their surrounding and can only be segmented by considering their larger context and shape. Such a situation is exactly where random forest classifiers typically fail, and indeed, the classical approach applied to this dataset resulted in a poor quality segmentation (Dice score of 0.33). In comparison, FeatureForest leads to the correct segmentation of the mitochondria with few spurious segmented pixels (Dice score 0.83). As before, the results can be further improved by using our post-processing (Dice score 0.87). The distribution of Dice scores over the whole dataset (500 slices) further shows that FeatureForest enables segmenting the stack with high fidelity while the random forest classifier leads to poor quality results (see Fig. 2d), with mean and standard deviations of 0.74 ± 0.06 (FeatureForest), 0.78 ± 0.07 (FeatureForest + post-processing), and 0.30 ± 0.06 (random forest classifier). Here again, other metrics corroborate the Dice score (see Table 1 and Supplementary Fig. 2), with FeatureForest vastly outperforming the random forest classifier. FeatureForest SAM2-based post-processing slightly improves the metrics scores while yielding a better visual impression (see Supplementary Fig. 3) due to the smoothness of the resulting segmentation. Note that the random forest classifier produces a large amount of spurious segmented pixels, leading to a low precision (0.26 ± 0.09) compared to its recall score (0.38 ± 0.07).

Next, we compared segmentation performance on data from a different imaging modality and sample type. Figure 2e showcases the output of Labkit and FeatureForest on an H&E-stained human kidney tissue. This data contains specific blood vessel structures called glomeruli. In the example from Fig. 2e, the glomeruli are slightly darker than their surroundings and, most importantly, display a wide variety of textures. The random forest classifier is capable of approximately segmenting many glomerulus instances, but misses several of them and produces many spurious groups of segmented pixels (Dice score 0.52). Here again, FeatureForest correctly segments all structures, and its post-processing leads to smooth and complete segmented objects. The dataset was created by tiling a larger image, and some tiles are shown in Supplementary Fig. 4, including the recomposed image, showcasing the performance of FeatureForest. Computing the Dice score for each tile (Fig. 2f) leads to mean and standard deviations of 0.81 ± 0.04 (FeatureForest), 0.87 ± 0.04 (FeatureForest + post-processing), and 0.61 ± 0.11 (random forest). Across all metrics, FeatureForest surpasses the random forest classifier (Labkit). However, FeatureForest scores relatively low on the boundary F1 (0.63 ± 0.07) compared to the other metrics. Post-processing substantially increases the performance on that metrics (0.87 ± 0.06).

Because FeatureForest uses a random forest as a classifier on top of the foundational model features, FeatureForest can segment multiple classes at a time. To demonstrate this, in Fig. 2g, we segmented a mouse embryo imaged in label-free brightfield microscopy. While the cells at the center of the embryo have a vastly different texture from the rest of the image, the extraembryonic membrane of the embryo has spatially varying intensity due to shadowing and is closer to the uniform background texture. The random forest classifier performs well on the cell mass (Dice score 0.91), but is subpar on the extraembryonic membrane (0.70), leading to incomplete segmentation of the latter. Once again, the classical random forest method erroneously segments other structures in the image, leading to the same imbalance between precision and recall as before (0.59 ± 0.09 vs 0.75 ± 0.03, respectively), as shown in Table 1. FeatureForest produces an almost perfect segmentation with high Dice scores (0.99 for the cells, and 0.90 for the extraembryonic membrane). This is the case throughout all the test images (see Fig. 2h), with mean and standard deviations of 0.90 ± 0.01 (FeatureForest), 0.93 ± 0.01 (FeatureForest + post-processing), and 0.66 ± 0.05 (random forest classifier). Other metrics confirm the segmentation performance of FeatureForest on the extraembryonic membrane class (see Table 1 and Supplementary Fig. 2).

To further showcase multiclass segmentation, we also segmented a FIB-SEM dataset distinguishing 6 classes (endoplasmic reticulum, golgi, mitochondria, lysosomes, lipid droplets and nuclear envelope). FeatureForest correctly segments most objects in the images (see Supplementary Fig. 5), across a wide range of texture and shapes.

### Multiclass segmentation on large datasets

For complex datasets, as we have seen, the performance of classical random forest pixel classification can lead to unusable segmentation, as shown in Fig. 2c. When training deep learning networks is not possible due the ground-truth label generation requirement, FeatureForest provides a useful alternative to perform the segmentation.

This was exemplified in a recent study^26^, in which FeatureForest was used to segment organelles in a complex symbiotic interaction between eukaryotic cells. The data consisted of large resin-embedded FIB-SEM stacks representing a dinoflagellate cell (referred to as the host). This dinoflagellate species is known to acquire and hijack organelles from its algal prey (microalga *Phaeocystis antarctica*), including nucleus, plastids and mitochondria, and retain them over several months.

**Fig. 3:**
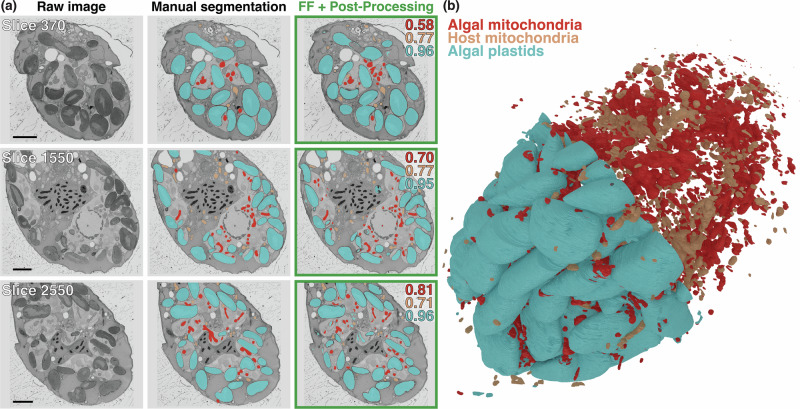
Segmentation of plankton organelles from a FIB-SEM stack using FeatureForest. **a** Three different slices (out of 3598) of a dinoflagellate cell imaged in FIB-SEM, overlaid with manual segmentation, and post-processed FeatureForest (*SAM2_Large*). The segmentation masks consist of three classes: algal plastids (blue), algal mitochondria (red) and host mitochondria (orange). Dice score between the ground truth and FeatureForest + post-processing is indicated on the top right corner for each class. Scale bar 4 μm. **b** 3D reconstruction of the three classes (algal plastids, algal mitochondria, and host mitochondria) of (**a**) throughout the entire dataset.

In Fig. 3a, we compare the manual segmentation of three classes (algal plastids, algal mitochondria, and host mitochondria) with the results from FeatureForest on three different slices of a single FIB-SEM stack from Rao et al.^26^ (original stack of size 3598 × 4455 × 3944 pixels, which was binned with a factor 4). The mitochondria of both the host (orange) and the algal prey (red) were segmented in two different classes in one FeatureForest model, while we trained FeatureForest again separately for the algal plastids (blue). In all cases, FeatureForest led to high-quality segmentation. In particular, the plastids are accurately segmented throughout the stack. To quantify this, we manually segmented 7 test slices distributed over the whole range of the stack. We then computed the Dice score between the manual segmentation and FeatureForest + post-processing on these test slices, confirming the visual impression, with mean and standard deviations of 0.58 ± 0.06 (algal prey mitochondria), 0.64 ± 0.03 (host mitochondria), and 0.88 ± 0.02 (algal plastids) (see Supplementary Fig. 6 for the distributions). Here, manually annotating 7 slices for quantification purposes was a slow process. In contrast, the trained FeatureForest classifier does not require additional inputs to segment the three classes in the 3598 slices of the entire stack. Segmentation of these organelles throughout such a large stack is essential to visualize and quantify morphological changes (e.g. changes in volume and surface of stolen organelles). The segmentation provided by FeatureForest allows building a 3D model of the distribution of organelles in space (see Fig. 3b), a necessary step in measuring the morphometrics of the various organelles. More details on the findings of the study are available in Rao et al.^26^.

### Comparing model performance

FeatureForest drastically improves segmentation quality compared to classical random forest-based approaches such as Labkit^3^, in particular on complex datasets such as the low-contrast electron microscopy dataset shown in Fig. 2c. In this section, we assess the performance of our FeatureForest approach when different feature-generating networks are used. Before, however, it is important to remind ourselves that the motivation to using our method is twofold: (*i*) the ease of use, even for users without any computational background or experience, and (*ii*) the iterative workflow, as described in Fig. 1, where a few scribbles by a user can already lead to initial results and any further labeling of pixels is guided by mistakes the current FeatureForest model makes. Other methods, like training a U-Net^27^, do not share these advantages, typically requiring some experience with setting up a deep learning training pipeline, and requiring so-called dense labels for every single pixel in the entire training set. As a comparison, we trained a U-Net^28^ for the Spheroid dataset, using 8 full slices of the available dense ground-truth labels, and observed that FeatureForest yields comparable performance to the trained U-Net (see Table 2 and Supplementary Figs. 7 and 8). While FeatureForest with post processing still outperforms the U-Net, the important insight is the relative ease at which users can achieve results as good as specifically trained neural networks on dense and perfect quality training data.

**Table 2:** Metrics score comparing a UNet, FeatureForest, and FeatureForest with post-processing

Method	Dice score *↑*	Precision *↑*	Recall *↑*	Boundary F1 *↑*	Hausdorff dist. *↓*
U-Net	0.78 ± 0.07	0.82 ± 0.08	0.75 ± 0.09	0.77 ± 0.07	10.16 ± 14.80
FeatureForest (FF)	0.74 ± 0.06	0.76 ± 0.09	0.73 ± 0.06	0.71 ± 0.07	10.80 ± 7.74
FF + Post-Processing	0.78 ± 0.07	0.78 ± 0.10	0.80 ± 0.06	0.79 ± 0.07	9.14 ± 8.27

The metrics are computed on the Spheroid (Fig. 2c, *N* = 512) dataset. The best performing method for each metric is underlined, where for Dice, precision, recall, and boundary F1 larger values are better (*↑*), while for the Hausdorff distance, smaller is better (*↓*). FeatureForest was trained using *SAM2_Large*.

So far, all the results were obtained using SAM2 (*SAM2_Large* model), the most powerful model currently available in FeatureForest. By default, MobileSAM^22^ and DINOv2^19^ can also be used to extract feature vectors. Other proposed methods that are similar to FeatureForest^29,30^ use a pretrained VGG16^31^ to generate features. Since our experiments did not display convincing results using VGG16 features, we do not offer this relatively outdated model in our own implementation. In Table 3, we computed all aforementioned metrics on the two electron microscopy datasets from Fig. 2 for various feature-generating models (see Supplementary Fig. 9 for the distributions). On the Fly brain dataset (see Table 3 and Supplementary Fig. 10), DINOv2 outperforms the other approaches, with SAM2 being a very close contender. MobileSAM, the smallest model available in FeatureForest, provides inferior performance to the larger networks, but fares better than VGG16. Visually, VGG16 over-segmented the image, yielding large false positive areas (Supplementary Fig. 10). This leads to results even inferior to those of the random forest classifier. On the more complex dataset (see Table 3 and Supplementary Fig. 11), the Spheroid dataset, SAM2 is the best model, with all others displaying similar, but inferior, performances. Overall, SAM2 provides reliable segmentation results, while the other models experience higher sample-dependent variability: they can perform well on a particular dataset and poorly on others.

**Table 3:** Quantitative comparison of random forest and FeatureForest using various models for feature vectors extraction

Dataset	Method	Dice score *↑*	Precision *↑*	Recall *↑*	Boundary F1 *↑*	Hausdorff dist. *↓*
Fly brain	Random Forest	0.61 ± 0.07	0.60 ± 0.09	0.62 ± 0.07	0.38 ± 0.07	11.54 ± 4.72
	VGG16	0.32 ± 0.10	0.25 ± 0.09	0.50 ± 0.16	0.17 ± 0.05	16.88 ± 6.52
	MobileSAM (FF)	0.69 ± 0.08	0.69 ± 0.10	0.70 ± 0.08	0.41 ± 0.07	9.79 ± 4.56
	DINOv2 (FF)	0.89 ± 0.04	0.88 ± 0.04	0.91 ± 0.06	0.76 ± 0.07	1.49 ± 0.94
	SAM2_Large (FF)	0.88 ± 0.05	0.91 ± 0.05	0.86 ± 0.06	0.70 ± 0.06	2.82 ± 2.04
Spheroid	Random Forest	0.30 ± 0.06	0.26 ± 0.09	0.38 ± 0.07	0.26 ± 0.08	73.63 ± 26.89
	VGG16	0.53 ± 0.08	0.43 ± 0.09	0.70 ± 0.09	0.42 ± 0.08	25.48 ± 11.92
	MobileSAM (FF)	0.57 ± 0.07	0.54 ± 0.10	0.61 ± 0.07	0.46 ± 0.07	29.85 ± 13.60
	DINOv2 (FF)	0.56 ± 0.07	0.53 ± 0.08	0.61 ± 0.08	0.49 ± 0.06	28.61 ± 11.41
	SAM2_Large (FF)	0.74 ± 0.06	0.76 ± 0.09	0.73 ± 0.06	0.71 ± 0.07	10.80 ± 7.74

The metrics are computed on the Fly brain (Fig. 2a, *N* = 256) and Spheroid (Fig. 2c, *N* = 512) datasets. *MobileSAM*, *DINOv2*, and *SAM2_Large* are available within FeatureForest (FF), while VGG16 is available within Convpaint^30^. Best performing method for each metric is underlined, where for Dice, precision, recall, and boundary F1 larger values are better (*↑*), while for the Hausdorff distance, smaller is better (*↓*).

As described previously, FeatureForest post-processing improved the results obtained with *SAM2_Large* in Fig. 2. We observed improvements for every model available in FeatureForest (see Supplementary Table 1 and Supplementary Fig. 12), confirming the utility of this feature.

To further test the robustness of FeatureForest against low contrast or low signal-to-noise ratio, we corrupted the Spheroid dataset and trained FeatureForest (*SAM2_Large*) with and without post-processing on the degraded images. Low contrast affects the dynamic range of the pixel values, while maintaining the integrity of the structures (see Supplementary Fig. 13). FeatureForest proved to be resilient to decreasing contrast, with performance degrading across all metrics for the lowest contrast level only, at which point structures were in fact barely visible any longer. We then generated low signal-to-noise ratio images using two different approaches (see Methods for descriptions): additive Gaussian noise (see Supplementary Fig. 14) and rescaled Poisson noise (see Supplementary Fig. 15). As opposed to lowering contrast, noise distorts the boundaries of objects in the image, complicating the segmentation task. In both cases, the performance across the various metrics decreases with the amount of noise (Supplementary Figs. 14 and 15). To allow for comparison, we estimated the signal-to-noise ratio (SNR) of the images degraded by both noise processes. FeatureForest segmentation quality, as measured by Dice score is equally sensitive to both degradation (see Supplementary Fig. 16).

### Training and prediction timing

The time required for each step of the FeatureForest pipeline varies, depending largely on the size of the training images, the chosen model, and the computer system. In particular, extracting the feature vectors is a lengthy operation that obviously scales with the size of the dataset. We measured the duration of the feature vectors extraction and writing to the HDF5 storage for a single slice of various sizes and for each model, on different operating systems, GPU's and CPUs (see Supplementary Fig. 17 and Supplementary Table 2). The extraction time is roughly linear with the input size. DINOv2 proved difficult to run on Windows due to missing optimization libraries. In every system we tested, the lightweight MobileSAM led to faster extraction time (ranging from 0.67 s to 1.77 s to extract a 256 × 256 slice on GPU) than the other models, and was even faster on CPU (1.61 s and 2.83 s) than the other models on GPU. Extraction time for *SAM2_Large* seemed to be dependent on the available GPU memory (going from 21.91 s on Windows with 6 GB GPU to 4.61 s on Linux with 32 GB GPU to extract a 256 × 256 slice). Finally, DINOv2 was performing well only on Linux systems. Note that the numbers reported here are indicative and will depend on a variety of factors, including operating system, NVIDIA drivers, specific GPU model, and installed library versions.

FeatureForest training steps consist of iteratively labeling the data, training the random forest, and predicting on the sample. In order to assess the duration of these various steps, we tracked the number of random forest trainings as a proxy for the number of iterations, as well as estimated the total training time by measuring the interval between the first and last labeled pixels (see Table 4), while training on the datasets from Fig. 2a (Fly brain) and 2c (Spheroid). These constitute imperfect measurements, since they include a number of iterations and predictions that are not reproducible across datasets and models, or between users. In addition, a model may not show signs of further improvement after fewer iterations than a better-performing model that requires more iterations to reach satisfying results. They nonetheless provide indications on the amount of time necessary to train FeatureForest on these datasets. In addition, we report the average training step and the slice prediction durations (see Table 4). Since training the random forest scales with the number of labeled pixels, the training time increases throughout the iterations as users tend to add labels rather than delete them. Single-slice prediction duration, on the other hand, only depends on the number of extracted features and model implementations, and is therefore stable throughout training. The Fly brain dataset required about 30 min to be trained on for each model, with DINOv2 converging rapidly (33 iterations) comparatively to the other models, all the while also taking the longest total time to train (43 min). The reason for the DINOv2 training being slower although consisting of fewer iterations was to be found in its slower prediction time on this particular dataset. SAM2, although a larger model than MobileSAM, trained in 20 minutes thanks to rapid convergence towards high-quality segmentation. The Spheroid dataset is more complex and required more iterations for DINOv2 and SAM2, but also longer training time for all models. In part, this is due to a much longer slice prediction time caused by larger image dimensions (each slice of the Spheroid dataset is 1024 × 512 compared to 256 × 256 in the case of the Fly brain). In addition, the dataset complexity also led to a higher number of labeled pixels, yielding longer average training steps. Note that single-slice prediction durations are smaller in Table 4 than the extraction durations reported in Supplementary Table 2 for the same image size, as the prediction step does not include writing the features to the feature vectors storage.

**Table 4:** Duration of the various steps in the FeatureForest training for two datasets

Dataset	Model	Iterations	Tot. training (min)	Average training step (s)	Slice prediction (s)
Fly brain	MobileSAM	55	33.96	5.46	1.49
13 × 256 × 256	DINOv2	33	43.58	3.71	4.48
	SAM2_Large	39	23.64	3.08	1.52
Spheroid	MobileSAM	49	110.52	9.43	21.17
17 × 1024 × 512	DINOv2	48	133.63	10.64	24.15
	SAM2_Large	60	141.35	12.53	29.50

The number of iterations is estimated as the number of random forest trainings performed. The total training time was measured as the interval between the first and last pixel labeling. The average training step was computed from averaging every random forest training. The prediction step duration for a single slice is constant, a single slice of the Fly brain being 256 × 256, while a single slice of the Spheroid is 1024 × 512. The size of the training stack is indicated under the dataset name. The measurements were performed on a Linux machine with a high-end GPU (NVIDIA A40-16Q, 16 GB).

Finally, once trained, the total prediction time required depends on the system hardware, operating system, and installed libraries. For the two datasets examined in this section, the whole stack prediction duration are estimated from Table 4 and reported in Table 5 for our Linux test system (16 GB GPU). In the case of the Fly brain, both MobileSAM and SAM2 are reasonably quick, being able to predict on the whole stack within 6 minutes. DINOv2, on the other takes a little under 20 minutes. The larger Spheroid stack (500 × 1024 × 512) leads to time scales in the order of a few hours, with both MobileSAM and DINOv2 predicting within 4 hours. As opposed to the other dataset, SAM2 was here the slowest model for prediction (4 hours and 10 minutes).

**Table 5:** Duration of whole stack prediction

Dataset	Model	Prediction (min)
Fly brain	MobileSAM	6.36
256 × 255 × 255	DINOv2	19.11
	SAM2_Large	6.49
Spheroid	MobileSAM	180.65
500 × 1024 × 512	DINOv2	206.08
	SAM2_Large	251.73

The reported values are estimated from Table 4 and correspond to prediction performed on a Linux machine with a high-end GPU (NVIDIA A40-16Q, 16 GB).

### Computational cost and minimum hardware requirements

The use of large foundation models in FeatureForest imposes constraints on the computational hardware required to process images efficiently. In particular, as with most deep-learning based tools, it is strongly recommended to use GPU-acceleration. The available memory in the GPU restricts which models can be used, as shown in Table 6. There, we estimated a loose minimum constraint on the GPU memory that allows running the model successfully with a 512 × 512 image. The GPU memory footprint will increase with larger images and with the number of slices in a stack. For small GPU units (< 4 GB), only MobileSAM can run. Larger GPUs (>= 6 GB) should be able to run *SAM2_Base* or *SAM2_Large*. For instance, our Windows test laptop with 6 GB GPU successfully extracted features of large images with *SAM2_Large* (see Supplementary Fig. 17). This is indicative as it heavily depends on the operating system, the GPU model, the specific driver, and the installed library versions.

**Table 6:** GPU memory and storage space requirements for different feature generating models

Model	GPU memory (GB)	Storage space (GB)	Storage space (GB)
	512 × 512	512 × 512	256 × 512 × 512
MobileSAM	3	0.352	42.539
DINOv2	10	0.213	48.470
SAM2_Base	6	0.844	102.094
SAM2_Large	8	0.844	102.094

The minimum GPU memory was estimated from the GPU memory footprint of running the model with a 512 × 512 image on Linux. The storage space corresponds to the feature vectors storage footprint on disk for 512 × 512 and 256 × 512 × 512 images.

Another constraint resulting from the training process of FeatureForest is the size of the image feature vectors storage. In Table 6, we show the memory footprint of the disk of the feature vector HDF5 storage for the different FeatureForest models. The size of the feature vectors is dependent on the chosen model, with a total storage space ranging from about 200 MB (DINOv2) to 800 MB (SAM2) for a 512 × 512 sized image. The total storage size scales linearly with the input shape, and a 256 × 512 × 512 image stack will require 50 GB disk space for MobileSAM and DINOv2, and 100 GB for SAM2.

Finally, training on and processing images with FeatureForest is barely influenced by the amount of random-access (RAM) memory, or number of CPU cores. FeatureForest only loads in RAM the images used for training or prediction, and does not use intensive multi-threading. In addition, most computers with a dedicated GPU come with enough RAM and CPU cores to be able to run standard image processing tasks, making them compatible with FeatureForest.

## Discussion

In this manuscript, we introduced FeatureForest, an approach leveraging existing foundation models to generate pixel-wise high-quality feature representations that are then used to train a random forest for pixel classification. Via our napari plugin implementation, FeatureForest provides a simple, intuitive, and straightforward segmentation pipeline, combining the power of large deep learning image segmentation models with the ease of use of random forests. Crucially, these models can be applied even by researchers with no knowledge of deep learning. FeatureForest fills a gap in the landscape of segmentation tools, in particular for large and complex datasets such as electron microscopy volumes, for which the annotation effort required to assemble ground-truth for deep-learning is considerable. We provide several different foundation models for feature generation, including SAM2, the current state-of-the-art large foundation model for segmentation, as well as the possibility for users to add their own model adapter to FeatureForest. Moreover, we also designed post-processing steps allowing further improvement of the results by using FeatureForest segmentation output to directly generate SAM2 predictions.

We benchmarked FeatureForest on multiple publicly available datasets that were published with ground-truth (or for which we could generate our own ground truth), including FIB-SEM, H&E stainings, and label-free brightfield images, both for single and multi-class segmentation. We showed that not only does FeatureForest significantly improve segmentation performances on these datasets compared to a classical random forest pixel classifier, but that it also produces high-quality segmentation for complex datasets for which the random forest classifier pixel results are unusable.

Here, we investigated FeatureForest with different models: MobileSAM, DINOv2, and *SAM2_Large*. *SAM2_Large* was the best performing model overall, only surpassed metrics-wise by a small margin by DINOv2 on a single dataset. DINOv2 otherwise underperformed on the other datasets. We therefore recommend using SAM2 models whenever possible as it delivers the best and most consistent segmentation quality. In cases where the available GPU is limiting, we suggest users to use FeatureForest with *SAM2_Base* first, followed by MobileSAM.

FeatureForest post-processing consistently improved results, leading to smoother masks and more complete objects. In certain cases, post-processing with bounding box generation can lead to over-segmentation when object instances are difficult to separate, and even in rare cases to a mask covering the entire image. In such cases, users might need to post-process these images separately with different parameters (e.g. smaller or larger number of smoothing steps).

We also showed that FeatureForest produced results of comparable quality to a well-trained deep-learning network, while not requiring dense ground-truth labels and offering the intuitive iterative learning approach presented in Fig. 1. We believe this is precisely this iterative labeling workflow that renders FeatureForest such a practical tool for life science users. Indeed, with such a workflow, they no longer need to densely label an a priori unknown amount of data, but are iteratively guided to locations where FeatureForest predictions are wrong, can relabel some pixels in these areas, and naturally stop this process when the results are of sufficient quality for further downstream analysis.

Our method inherits some inconveniences that are inherent to the large deep-learning models we use to extract feature vectors. Firstly, SAM2, MobileSAM, and DINOv2 are trained on natural images (e.g., scenes of everyday life, often RGB images) and the feature vectors they produce might not be optimized for separating the biological objects of interest. To address this, fine-tuning these models on microscopy images is an exciting possibility that the community is now starting to explore^13–17^. We also provide a preliminary integration of models from μSAM^16^. These are domain-specific models that finetune SAM for light and electron microscopy data and that may lead to even further improved semantic segmentation results for these domains.

A further limitation concerns the storage and generation of feature vectors. In order to be time-efficient, feature extraction should preferentially be performed on a graphical processing unit (GPU). Without access to a GPU, users should expect the feature extraction and the segmentation of full stacks for which features were not pre-exported, to take from minutes to hours, depending on the stack size. As this is the most time-consuming step, we separated the feature extraction and training steps in our napari plugins. Once FeatureForest is trained, the features are computed on the fly while segmenting an entire dataset. We therefore advise users to train on a representative sub-stack of the image in order to minimize the footprint on disk and generation time of the feature vectors, and segment on the larger stack once they are satisfied with the results on the training stack. In addition, the more complex models have a larger memory footprint as they consist of a much larger number of parameters. Future updates may include further optimization of memory usage, such as pruning the feature vectors from non-essential features and improving GPU usage.

During the work on FeatureForest, similar approaches have been co-developped, highlighting the usefulness of the method^29,30,32,33^. Compared to these variants, we use state-of-the-art foundation models to generate the feature vectors (e.g. MobileSAM, SAM2), rather than simpler and older networks such as VGG16^31^ or networks trained on very specific datasets. Compared to SAM2, VGG16 has the advantage of being lightweight, and therefore able to run on most machines. In our experiments, however, VGG16 did not provide substantial improvements over classical random forests and was, as expected, vastly inferior to using SAM2 features.

In the future, we will continue to optimize FeatureForest in order to further improve the user experience, in particular with respect to speed and memory efficiency, and by adding more models for feature extraction and post-processing. The source code for our napari plugins is freely and openly available on Github^34^, and can be installed through PyPI. We also provide documentation on how to use FeatureForest, as well as scripts, notebook examples, and command-line interface for running FeatureForest outside napari (e.g., on high-performance computing (HPC) systems). We believe that FeatureForest constitutes a much-needed tool for many studies that deal with complex images.

## Methods

### FeatureForest napari plugins

FeatureForest is a Python software package and consists of convenience functions and a napari plugin. All code and documentation is accessible on Github (juglab/featureforest). The FeatureForest napari plugin contains two different widgets: *Feature Extraction* and *Segmentation widget*. The first plugin extracts feature vectors for each pixels in a selected napari layer and stores them in a HDF5 container to allow random access. The second widget allows training the random forest classifier using the previously exported feature vectors, as well as performing post-processing and segmentation of the entire dataset.

### Models

The embeddings of deep-learning networks are often of smaller spatial dimension that those of the input images, while having many more channels (the features). In order to obtain per image pixel features, we split the images into overlapping patches. The constraints on the patch size and on the overlaps are model-dependent, and described below. Next, we upscale the patches to fit the model input size using bicubic interpolation (*Resize* from the *torchvision.transforms.v2* module). Since the models require RGB input, the single-channel patches are duplicated and concatenated into 3-channel patches. We apply the model to the patches, and save the resulting embeddings. Typically, these embeddings are the output of the encoder part of the model. See the various descriptions that follow for model-dependent details. Those embeddings have spatial dimensions smaller than that of the original patches, and we therefore upscale them again using bicubic interpolation, as described earlier. Using small input patches reduces the scale of the embeddings upscaling, leading to more distinctive features between neighboring pixels. Finally, the embedding patches, now of the same size as the patch inputs, are cropped to the non-overlapping regions and re-assembled as a feature map of the same spatial extent as the original image with *N* channels corresponding to the features, *N* being dependent on the specific model used (see below).

FeatureForest includes the following models that were used in this manuscript: *SAM2_Large*^23^, *SAM2_Base*^23^, *MobileSAM*^22^, and *DINOv2*^19^. All models are implemented by extending the *BaseModelAdapter* class, which allows setting a patch size compatible with the specific model, as well as extracting feature vectors for each pixel provided to the model. Each model has its own implementation, as they have different input requirements and architectures.

More specifically, *SAM2_Large* uses “*sam2.1_hiera_large.pt*” as a model, while *SAM2_Base* corresponds to the lighter “*sam2.1_hiera_base_plus.pt*” (see facebookresearch/sam2 on Github). We chose a maximum patch size of 512 and a minimum number of patches per dimension of 2. If images are smaller than half the patch size, we halve the patch size iteratively until the patch size meets the constraint of at least 2 patches per dimension. The overlap is chosen as half of the patch size. Patches are scaled to 1024 × 1024, SAM2 input dimensions. SAM2 encoder includes a Feature Pyramid Network (FPN, *backbone_fpn*) that outputs embeddings at three distinct resolution levels (64 × 64, 256 × 256, and 128 × 128). We independently upscale these embeddings to the patch size and concatenate them, leading to 768 features per pixel.

*MobileSAM* model uses a modified version of the *TinyVIT* model architecture that gives access to the internal embeddings computed by the encoder. We use “*mobile_sam.pt*” (see ChaoningZhang/MobileSAM on Github) as weights of our modified visual transformer architecture. We use the same patch and overlap constraints as for SAM2. *MobileSAM* encoder outputs 256 features. It also computes a 64 patch embeddings^35^ (*PatchEmbed* class), which are returned by our custom implementation of the encoder. We concatenate these embeddings to obtain 320 features in total per pixel.

Finally, we use “*dinov2_vits14_reg*” from the PyTorch Hub for *DINOv2*. DINOv2 input patches of size divisible by 14. To obtain per-pixel output, we create patches of fixed size 70 × 70 with overlaps 28 × 28. The number of output features for each pixel is 384, and is the output of the model itself.

For each experiment, FeatureForest was run from the commit *4aef995*, with the codebase being available on Github (juglab/featureforest). Unless otherwise indicated, the training and post-processing were carried out with defaults parameters. All training, analysis, and plotting were performed in Python using open-source libraries, using the GPU conda environment provided in the source code repository. Unless stated otherwise, all training and predictions were performed on a Linux virtual machine (RedHat) with access to a NVIDIA A40-16Q (16 GB) GPU using SAM2 model.

### FeatureForest random forest training

FeatureForest trains a random forest classifier using the feature vectors extracted from one of its adapted models. For each labeled pixel in the labeling layer in napari, the corresponding feature vectors are extracted, and fed along with the labels to the random forest classifier^36^. By default, we use 450 trees of maximum depth 9. The trained classifier can then be used to predict pixel label class for each pixel in the image or slice currently displayed in napari, or predict on the whole stack.

### SAM2 post-processing

As part of FeatureForest pipeline, we provide several post-processing options that leverage the large deep learning network used for feature generation. In any case, the first step employs mean curvature smoothing, an iterative edge-preserving smoothing method that fill small holes, and filters out small connected components. Users can change the number of smoothing iterations and the threshold used to filter out connected components by area (absolute or relative). By default, we use 25 smoothing iterations, and an absolute threshold of 50 pixels.

Subsequently, users can use either of the two additional steps: *SAM2ImagePredictor* and *SAM2AutomaticMaskGenerator*. In the former, we use a watershed algorithm to separate the mask into instances. Bounding boxes are then generated around each instance, and used as prompts for SAM2. The output instances are merged into a single mask and added into napari as a layer. *SamAutomaticMaskGenerator* generates an evenly-spaced grid of points as prompts to SAM2, which outputs a large number of masks. We retain only instances with an intersection over union, with respect to the closest connected component from the random forest segmentation, larger than a user-set threshold (by default 0.35).

### Datasets

The Fly brain dataset from Fig. 2a and Supplementary Fig. 1 is available as part of the *EMPIAR-10982* dataset, and consist of a stack of size 256 × 255 × 255 and an isotropic pixel size 12 nm. We use every 16 frames, starting from the first one, as training set, while prediction was performed on the whole dataset. In the figures, only images that were not used for training and are as far as possible from neighboring training slices are shown.

The human breast cancer spheroid stack (Fig. 2c and Supplementary Fig. 3) is extracted from *EMPIAR-11380* (sample *F059_bin2*)^37^. The stack has dimensions 1446 × 1683 × 1928 and an isotropic pixel size of 20 nm. We cropped the data to size 500 × 512 × 1024 from the top-left coordinate (390, 800, 150). We report slice numbers from the original dataset rather than from our cropped stack. Training was performed using every 30th frame, starting from the first, while prediction was performed on the whole dataset. In the figures, only images that were not used for training are shown, selecting specifically slices that are as far as possible in z from the training slices. For noise and contrast experiments, we made a test substack of 10 slices (405, 455, 505, 555, 605, 655, 705, 755, 805, and 855 indexed in the original stack).

The human kidney tissue example (Fig. 2e and Supplementary Fig. 4) is part of a dataset that was compiled from the Human Biomolecular Atlas Program (HuBMAP) and publicly released as part of a Kaggle challenge (www.kaggle.com/c/hubmap-kidney-segmentation/data). Specifically, we selected the *1e2425f28* sample, and used the fourth series (resolution 4027 × 3347), and cropped it to 1024 × 3072 (top-left coordinates (486, 1532)), before tiling it into a set of 512 × 512 images (*N* = 12). The masks were provided as instances in a json file and were converted into a binary image, before being cropped and tiled to match the raw image. We trained FeatureForest on the first four frames, and predicted on the whole tile stack.

The mouse embryo dataset (Fig. 2g) is publicly available on the Broad Bioimage Benchmark Collection with access number *BBBC003*. It consists of 5 slices of a 3D label-free brightfield stack of size 640 × 480 and pixel size 420 nm. As the initial ground truth only included the segmentation of the embryo as a single class, we manually labeled the extraembryonic membrane as a second class to generate two-label ground truth. Training was performed on the first slice, and prediction on the whole stack.

The U2OS FIB-SEM dataset (Fig. 5) is publicly available as *EMPIAR-11746*^38^, and consists of a 1168 × 3394 × 1385 stack with pixel size 2.5 nm in X and Y, and 0.5 nm in Z. We down-scaled the whole stack to a width of 1200, and used every 40 images starting from slice 500 as training dataset, and predicted on every 30 slice from slice 501 (test dataset). We used 6 out of the 8 classes available in the dataset ground-truth.

The dinoflagellate FIB-SEM dataset (Fig. 3) is part of a recent publication^26^ and is publicly available (EMPIAR-12627). It was high-pressure frozen and resin-embedded before imaging, and has dimensions 3598 × 4455 × 3944 pixels. More details about sample preparation are available in Rao et al. We binned the stack with a factor 4 (3598 × 1113 × 986 pixels) to work on a smaller stack. We used slices 50, 275, 462, 752, 1024, 1375, 1721, 2015, 2310, 2813, and 3067 for training and predict on every 3 slices (total number of 1200 slices). To allow for quantification, we manually labeled 7 slices (370, 650, 900, 1550, 1850, 2175, 2550, referred to as the test stack) with the three classes using the SAMJ ImageJ2 plugin (SAM2, segment-anything-models-java/SAMJ-IJ on Github).

### Metrics

We implemented Dice score, precision and recall calculations by counting true positive, false positive and false negative pixels while comparing ground truth masks and prediction results. Boundary F1 was adapted from Github (minar09/bfscore_python), and we used scikit-image’s^39^ implementation of the Hausdorff distance.

### Comparing Labkit and FeatureForest

In Fig. 2, for each dataset, we trained Labkit^3^ as the random forest classifier and FeatureForest using *SAM2_Large* on the training stack. Labkit was run independently by different image analysts, each analyst saved various classifiers with different sets of labels and filter standard deviation settings. We reported only the best performing classifier, evaluation of performances was carried out on the reported metrics. We used the default filters, and filter standard deviations [1, 2, 4, 8] (default values) for the Fly brain and Embryo, and [1, 2, 4, 8, 16, 32] for the Spheroid and Kidney dataset. Predictions were performed on the entire dataset for both Labkit and FeatureForest, and FeatureForest post-processing was carried out with default parameters. Panel (a), (c), (e), and (g) correspond to slice number 72 (Fly brain), 435 (Spheroid, cropped to a square region), 6 (Kidney), and 4 (Embryo). Dice scores in panels (b), (d), (f), and (h) are computed over the entire datasets (each pixel counting as true positive, true negative, or false negative).

To obtain Table 1 and Supplementary Fig. 2, we ran the various metrics for the same prediction results as in Fig. 2 over the entire stacks, and computed mean and standard deviation for each metrics and method (random forest, FeatureForest and FeatureForest with post-processing).

In Supplementary Figs. 1, 3 and 4, we show the prediction results of the model trained in Fig. 2. Dice scores were computed for each single slice against the ground-truth. In Supplementary Fig. 1, we show slices 40, 124, 168, and 232. In Supplementary Fig. 3, we used slices 435, 525, 705, and 885. Finally, in Supplementary Fig. 4, we show tiles 7, 8, and 9 in panel (a), while panel (b) shows an overlay of the ground-truth and the prediction for the entire untiled image.

### FeatureForest with multiple classes

In Supplementary Fig. 5, FeatureForest was trained with 6-class labels on the training stack, and prediction was performed on the entire stack. Slices 651, 891, and 1131 were slightly cropped to exclude white border without information.

In Fig. 3 and Supplementary Fig. 6, to obtain the segmentation of the three classes (host mitochondria, algal mitochondria, and algal plastids), we trained two different FeatureForest classifiers: one to segment the two types of mitochondria, and one to segment the plastids. Manual segmentation was performed using SAM2 (SAMJ plugin). The Dice scores were computed over the test stack. In order to visualize the segmentation in panel (b), we performed segmentation and post-processing of the three organelles using the classifiers trained for panel (a) on 1200 slices. The final post-processed stack was curated to re-segment with different post-processing parameters (smoothing steps 23 or 28 instead of the default 25) slices 263, 626, 628, 631, 690, 839, 859, 860, and 864 (out of the 1200 slices) to avoid whole-slice masks. The 3D reconstruction was performed in Blender 4.2 using MicroscopyNodes^40^.

### UNet training and comparison

We trained a 2D U-Net using BiaPy^28^ on 8 slices (390, 420, 450, 480, 510, 540, 570, and 600) for 100 epochs, with batch size 32, patch size 256, AdamW optimizer, initial learning rate 0.0001 and patience 20. We use the ground truth provided with the public dataset. Prediction was performed on our entire modified dataset (500 slices). We used the same FeatureForest results as in Fig. 2. SAM2 segmentation was obtained by manually prompting SAM2 using the ImageJ2 plugin SAMJ.

### Comparing models for feature extraction

In order to compare the various models, we retrained FeatureForest from scratch using MobileSAM and DINOv2 on the same training stacks as in Fig. 2, albeit with different labels, and predicted on the entire datasets (Fly brain and Spheroid). We computed the metrics as described above. Since FeatureForest does not include VGG16^31^, we used Convpaint^30^ (version 0.5.2) as a comparison, using the last layer of VGG16 as feature-generator (*features.28*), pyramid downscaling [1, 2], and 1 order of interpolation. We kept the default settings for the classifier. We showed the same slices in the figure as in Fig. 2.

For Supplementary Table 1 and Supplementary Fig. 12, we performed post-processing using default parameters of the FeatureForest models from Table 3.

### Low contrast experiment

We created four low contrast stacks by subtracting the mean value from the entire stack, multiplying by the contrast coefficient (0.8, 0.6, 0.4, and 0.2), and adding back the original mean to the stack: ${{\mathcal{I}}}_{c}=c\times ({{\mathcal{I}}}_{0}-\mu )+\mu$. This leads to an image stack of similar mean but smaller standard deviation. We trained FeatureForest on all stacks using the same labels as in Fig. 2. We computed the metrics over our test stack (10 slices). The reported slice is indexed as in the original stack.

### Low signal-to-noise ratio experiments

To generate the entire stack degraded with Gaussian noise, we added to each pixel a value drawn from a normal distribution of mean 0 and standard deviation 2, 10, 20, 50, and 100: ${{\mathcal{I}}}_{c}={{\mathcal{I}}}_{0}+{\mathcal{N}}(0,c)$. We trained FeatureForest on all stacks using the same labels as in Fig. 2. We computed the metrics over our test stack (10 slices). The reported slice is indexed as in the original stack.

Since Poisson noise is signal-dependent, we generated the Poisson noisy stack by dividing the pixel values by a coefficient (2, 10, 20, 50, and 100), drawing the new pixel value from a Poisson distribution parameterized by the result of the division, and multiplying again with the coefficient in order to recover the original mean: ${{\mathcal{I}}}_{c}={\mathcal{P}}({{\mathcal{I}}}_{0}/c)\times c$. We trained FeatureForest on all stacks using the same labels as in Fig. 2. We computed the metrics over our test stack (10 slices). The reported slice is indexed as in the original stack.

For each noise level of Gaussian and Poisson degradation, we computed the signal-to-noise ratio (SNR) as $10\times \log (\mu /\sigma )$, where *μ* and *σ* are the stacks mean and standard deviation, respectively. The Dice score means and standard deviations plotted are those measured over the test stack in Supplementary Figs. 14 and 15.

### Feature extraction duration

To estimate the time required to extract and write feature vectors to disk, we used a python script that creates a random image of specific size, instantiates one of the FeatureForest models and timed the feature extraction to disk using the corresponding internal FeatureForest function (*extract_embeddings_to_file*). We ran the script on several systems: Linux virtual machine (RedHat, with NVIDIA A40-16Q 16 GB GPU or in CPU mode), Linux HPC node (RedHat, with NVIDIA Tesla V100-SXM2-32GB GPU), Windows virtual machine (Windows 10 Enterprise, with NVIDIA A40-8Q 16 GB GPU), and a Windows laptop (Windows 10 Enterprise, with NVIDIA RTX-A3000 6 GB or in CPU mode). We estimated the average extraction time per 256 × 256 block by averaging the ratio between the extraction time and the number of pixels in the input, and scaling it again to 256 × 256 pixels.

### Timing labeling, training, and prediction

We implemented a timing statistics function in FeatureForest that logs the time interval between the first and last pixel being labeled ("Total training”), the number of calls to the random forest training ("Iterations”), the duration of each random forest training, and the prediction time for a single slice ("Slice prediction”). We recorded and compiled these measurements for the datasets and the FeatureForest models trained for Fig. 9 in Table 4. We averaged the duration of each random forest training to obtain the “Avg training step”.

The prediction time in hours reported in Table 5 were estimated from the number of slices in the entire datasets and multiplied with the single slice prediction time ("Slice prediction”) from Table 4, as the prediction time per slice is constant.

### Estimating GPU memory and feature vector storage

The minimum GPU memory requirements were estimated in the same script used for Supplementary Fig. 17. There, we systematically ran feature extraction with different models while restricting available memory using *set_per_process_memory_fraction from torch.cuda*. We determined the lowest memory allocation at which the model executed successfully, providing an estimate of the minimum GPU memory needed for the pipeline to run. The script additionally inspected the output file size of the feature vectors storage. We reported the values for 512 × 512 and 8192 × 8192 (the latter being equivalent to 256 × 512 × 512).

## Supplementary information

Supplementary Information

## Data Availability

All datasets are publicly available on EMPIAR (accession codes: 10982, 11380, 11746 and 12627), BBBC (BBBC003) and Kaggle (kaggle.com/c/hubmap-kidney-segmentation/data).

## Code availability

All source code is released under BSD-3-Clause license and freely available on Github^34^.
