# Supplementary material for: FeatureForest: the power of foundation models, the usability of random forests
Source: Npj Imaging. 2025 Jul 8;3:32. doi: 10.1038/s44303-025-00089-9 (PMC12238231; doi:10.1038/s44303-025-00089-9)
Supplement: Supplementary file 1 — Supplementary Information [file 44303_2025_89_MOESM1_ESM.pdf]

Supplementary Information

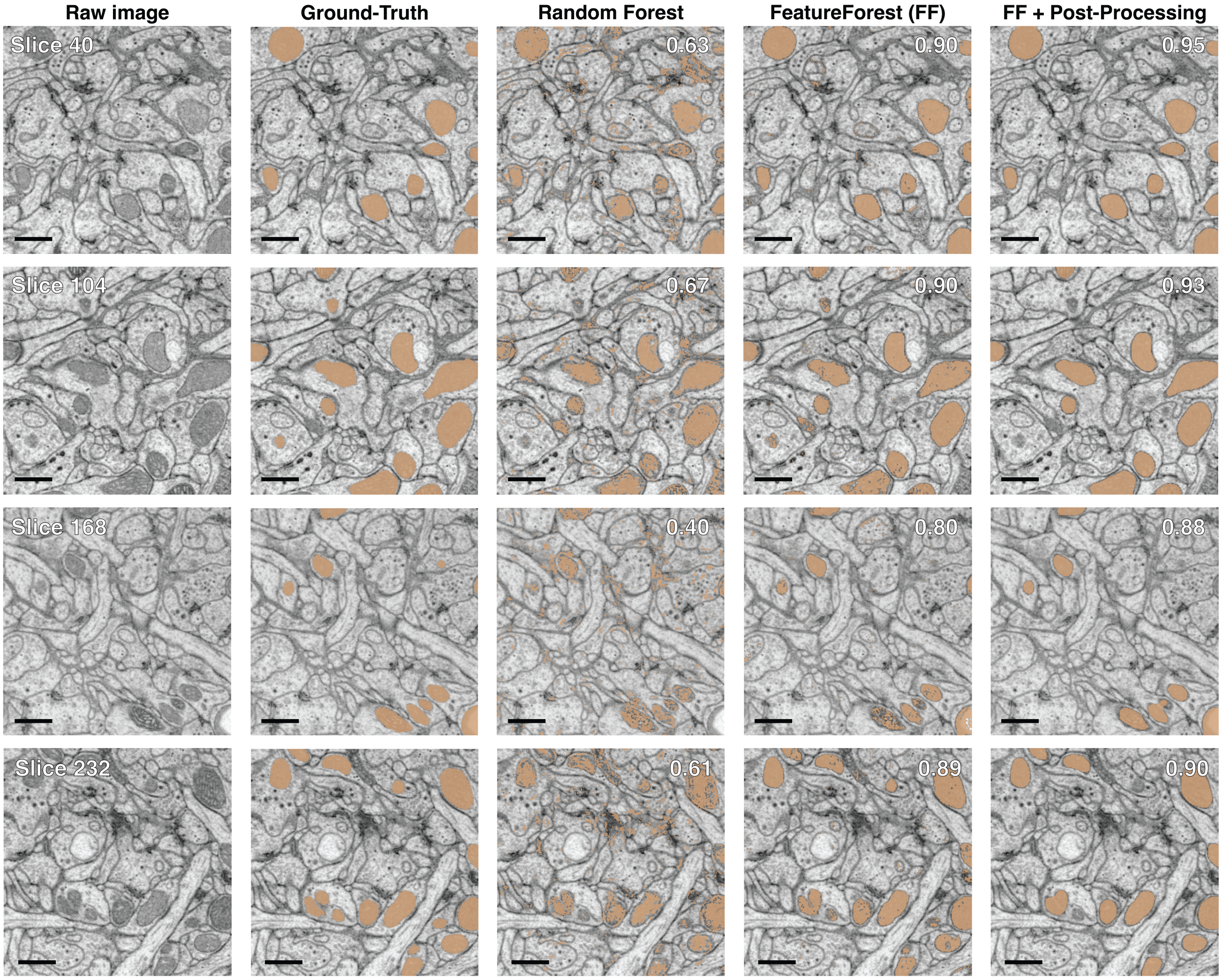

Supplementary Figure 1. Results on several slices of the FIB-SEM fly brain dataset. FIB-SEM images of a fly brain, overlaid with mitochondria ground-truth mask, and with corresponding segmentations obtained with a random forest classifier, FeatureForest, and after post-processing the results from FeatureForest, from left to right respectively. Slice number is indicated on the top left corner of the raw image, and the Dice score with respect to the ground-truth for the specific slice and algorithm is indicated in the top right corner. Scale bars 500 nm.

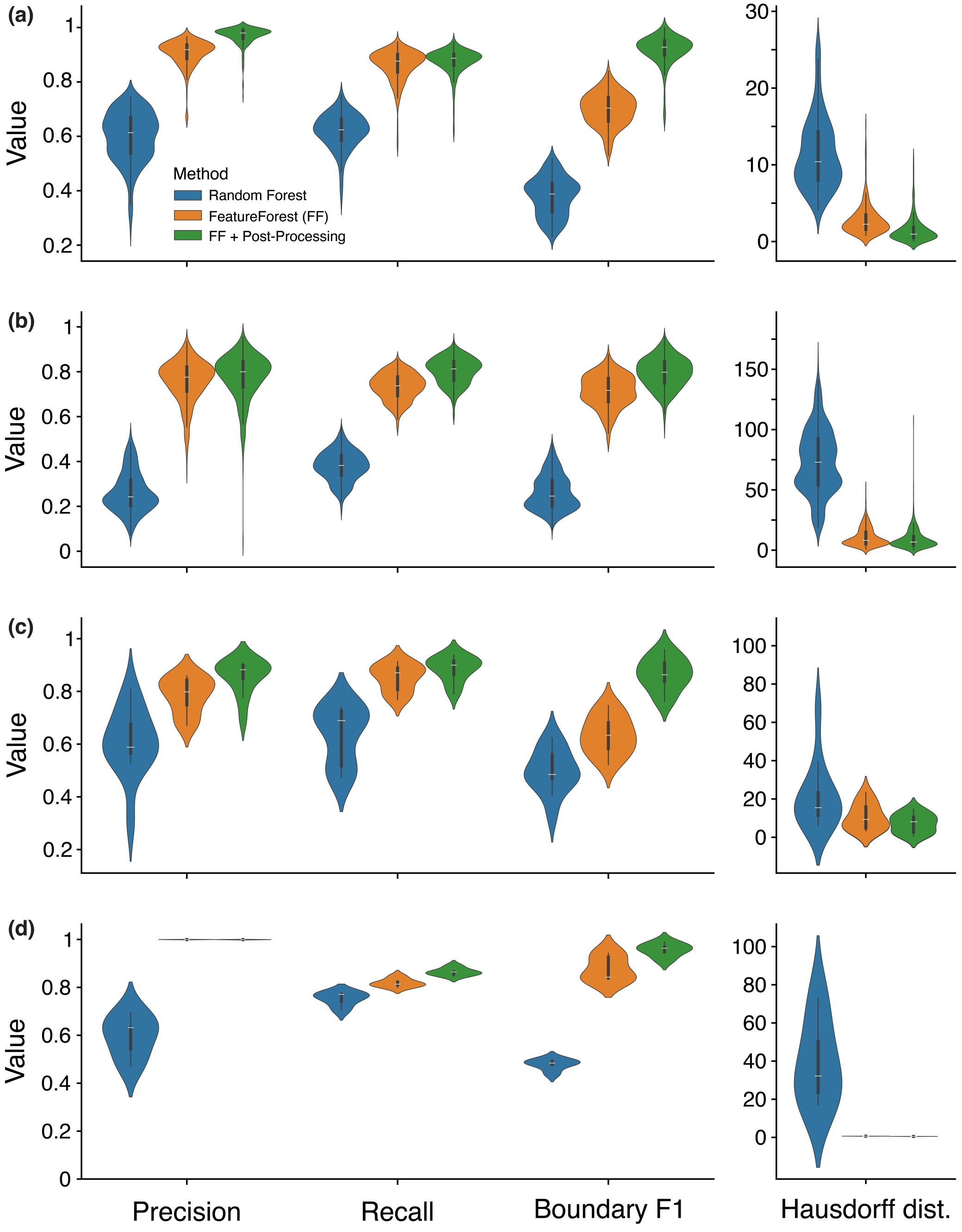

Supplementary Figure 2. Precision, recall, boundary F1 and Hausdorff distance distributions on the datasets of Fig 1. (a) Metrics measured on the Fly brain dataset for the random forest classifier, FeatureForest and FeatureForest with post-processing. Best performing methods reach higher values for precision, recall and boundary F1, and lower values for the Hausdorff distance. (b) Same for the Spheroid dataset. (c) Same for the Kidney dataset. (d) Same for the Embryo dataset (membrane class only).

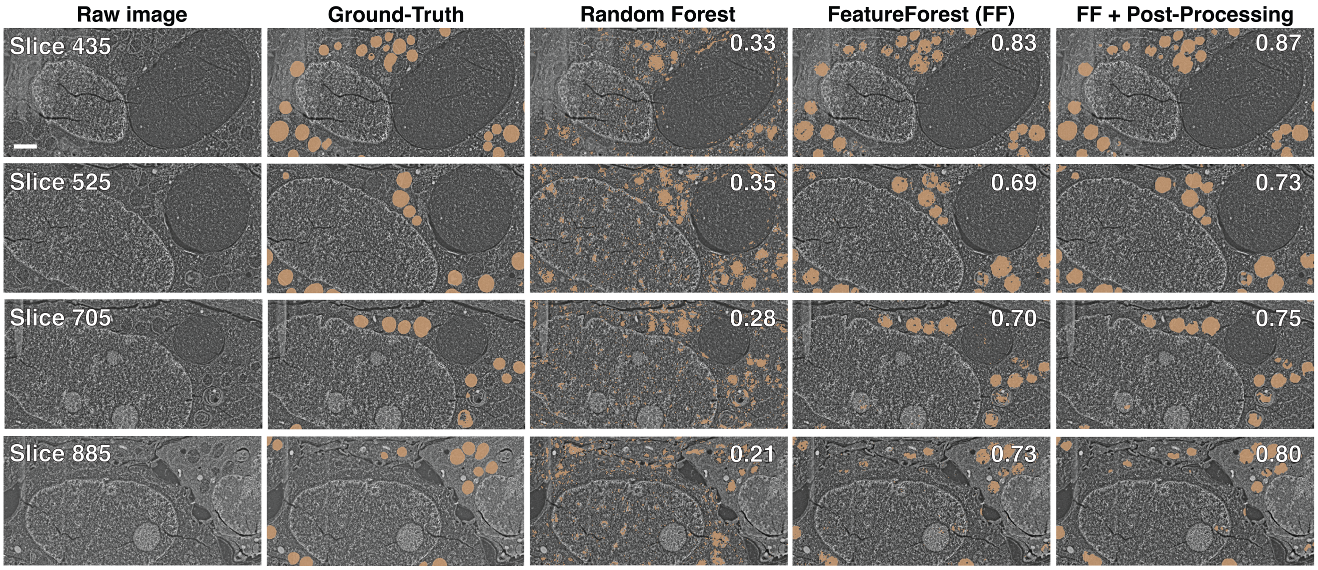

Supplementary Figure 3. Results on several slices of the FIB-SEM spheroid dataset. FIB-SEM images of a human breast cancer spheroid, overlaid with mitochondria ground-truth mask, and with corresponding segmentations obtained with a random forest classifier, FeatureForest, and after post-processing the results from FeatureForest, from left to right respectively. Slice number is indicated on the top left corner of the raw image, and the Dice score with respect to the ground-truth for the specific slice and algorithm is indicated in the top right corner. Scale bars 1 μm.

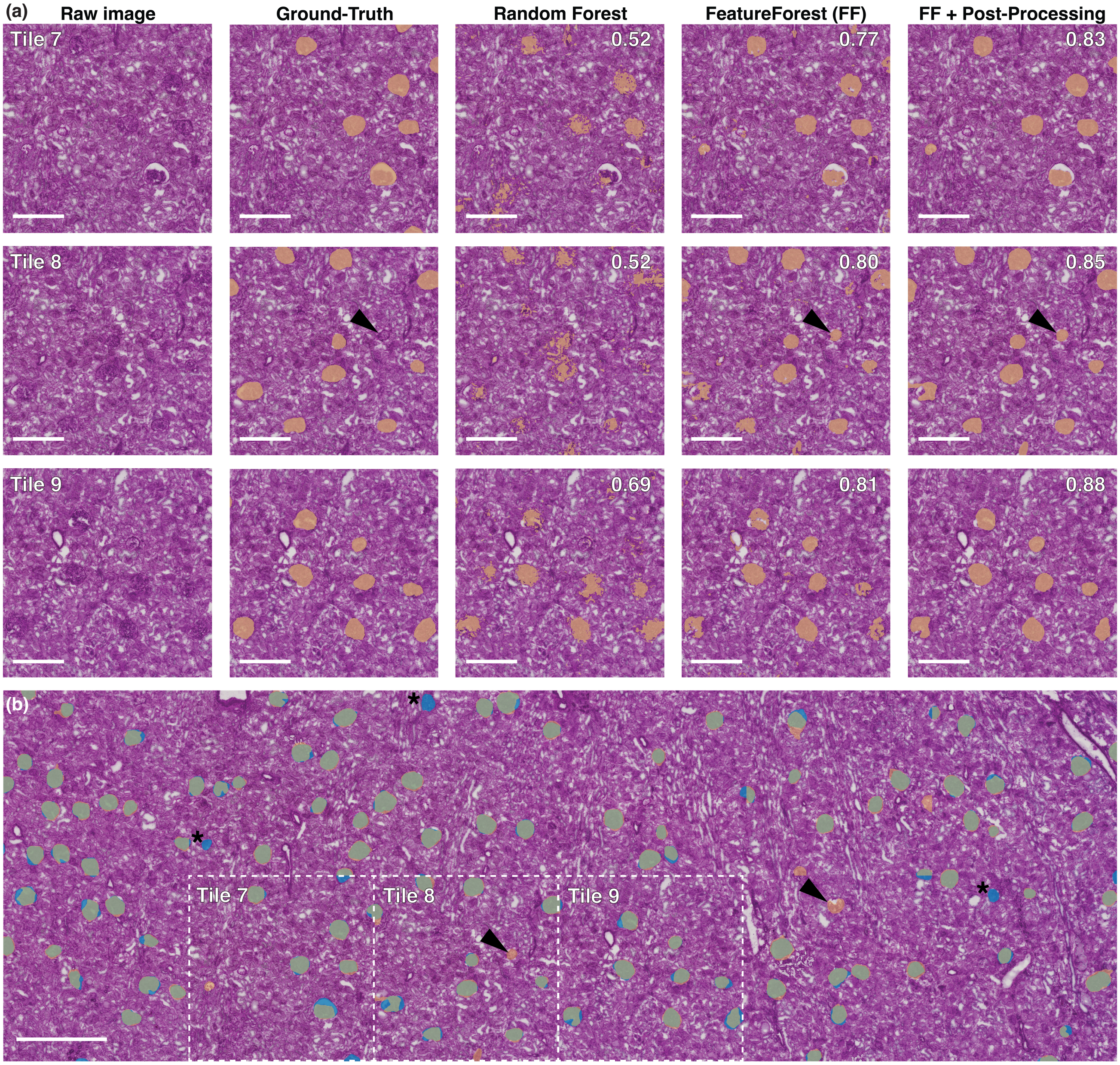

Supplementary Figure 4. Results on H&E stained human kidney dataset. (a) H&E staining of a human kidney, overlaid with glomerulus ground-truth mask, and with corresponding segmentations obtained with a random forest classifier, FeatureForest, and after post-processing the results from FeatureForest, from left to right respectively. Arrows indicate a glomerulus instance segmented by FeatureForest and absent from the ground-truth. Tile number is indicated on the top left corner of the raw image, and the Dice score with respect to the ground-truth for the specific slice and algorithm is indicated in the top right corner. (b) Larger image from which the tiles are extracted with an overlay of both the ground-truth (blue) and FeatureForest + post-processing (orange). As in (a), arrows indicate an error in the ground-truth that is correctly segmented by FeatureForest. Asterisks label instances that are present in the ground-truth and absent in the FeatureForest segmentation. Tiles of (a) are delimited by the dotted rectangles. Scale bars 500 µm.

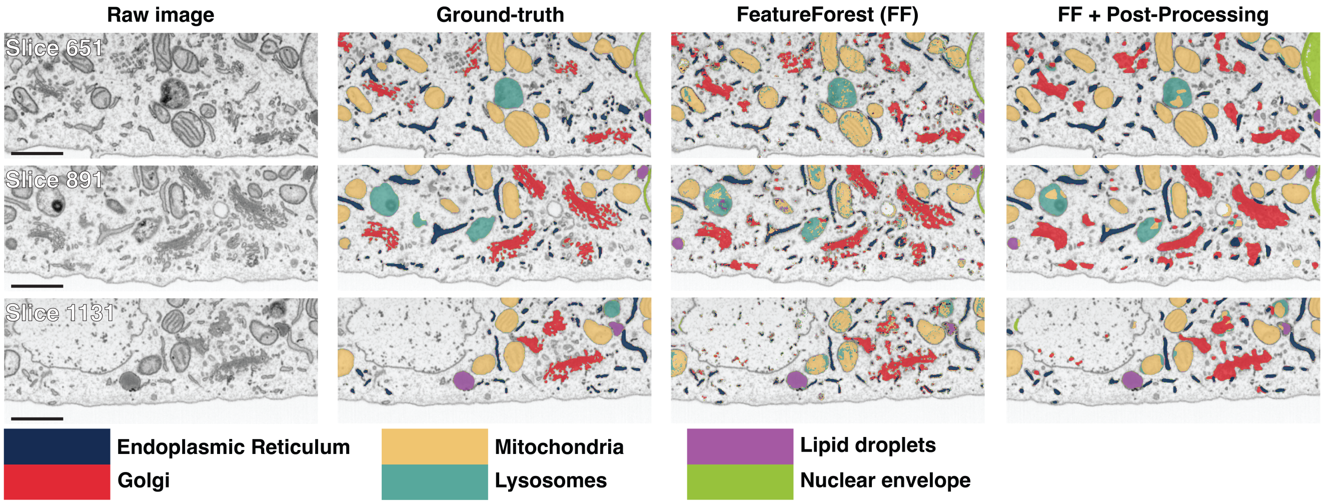

Supplementary Figure 5. Results on several slices of FIB-SEM images of a U2OS cell. FIB-SEM images of a U2OS cell, overlaid with ground-truth for 6 different classes (see legend), and with corresponding segmentation obtained with FeatureForest, and after post-processing the results from FeatureForest, from left to right respectively. Slice number is indicated on the top left corner of the raw image. Scale bars 1 µm.

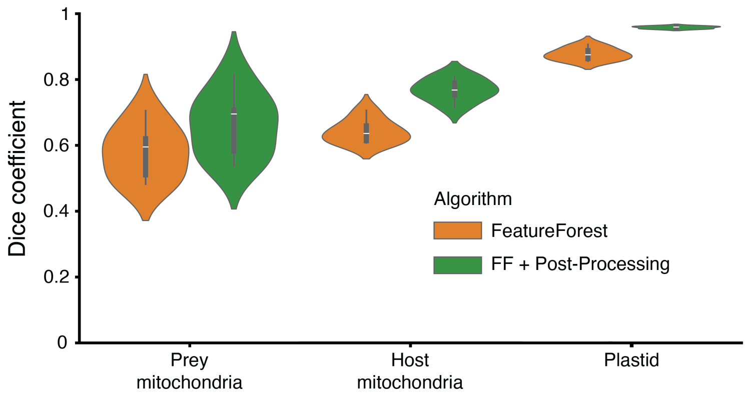

Supplementary Figure 6. Dice score over the test stack for the Dinoflagellate dataset. Distribution of the Dice score over the test stack (7 slices) for FeatureForest and FeatureForest with post-processing for the three organelle classes.

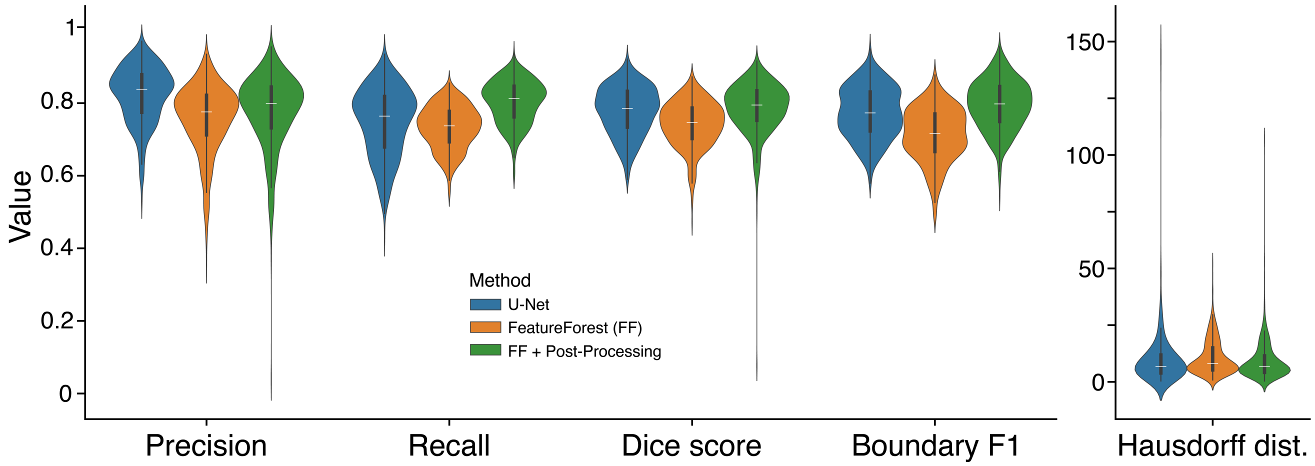

Supplementary Figure 7. Precision, recall, Dice score, boundary F1 and Hausdorff distance distributions for FeatureForest, FeatureForest + Post-Processing, and a U-Net trained on the Spheroid dataset. Best performing methods reach higher values for precision, recall, Dice, and boundary F1, and lower values for Hausdorff distance.

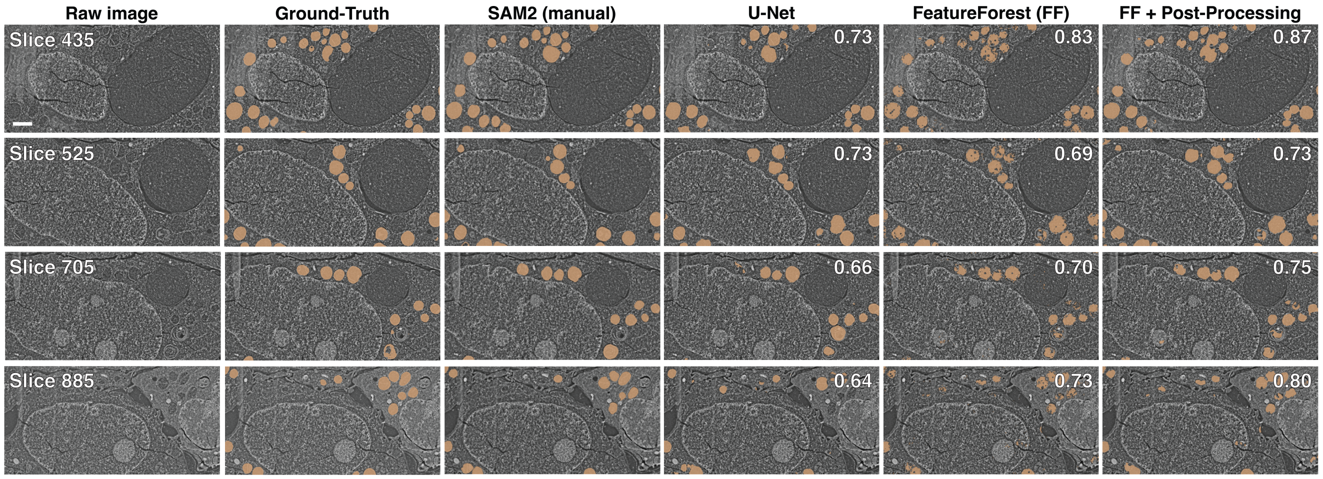

Supplementary Figure 8. Comparison of the segmentation performance of SAM2 with prompting, U-Net, FeatureForest, and FeatureForest with Post-Processing on the Spheroid dataset. Dice score for the particular slice and method is reported in the corner of each relevant image. Scale bar 1 µm.

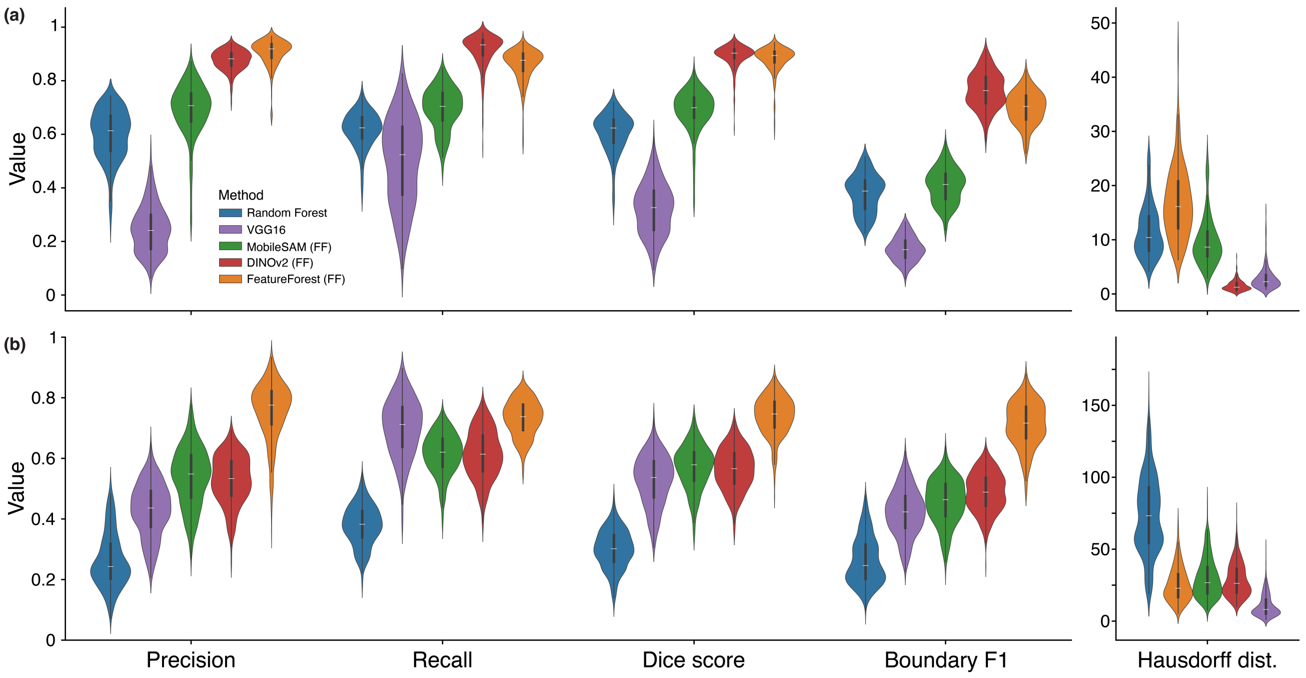

Supplementary Figure 9. Precision, recall, Dice score, boundary F1 and Hausdorff distance distributions for the different models on the Fly brain and Spheroid datasets. (a) Metrics measured on the Fly brain dataset for the random forest classifier, VGG16 (Convpaint), and models available in FeatureForest: MobileSAM, DINOv2 and SAM2 (*SAM2_Large*). Best performing methods reach higher values for precision, recall, Dice, and boundary F1, and lower values for Hausdorff distance. (b) Same for the Spheroid dataset.

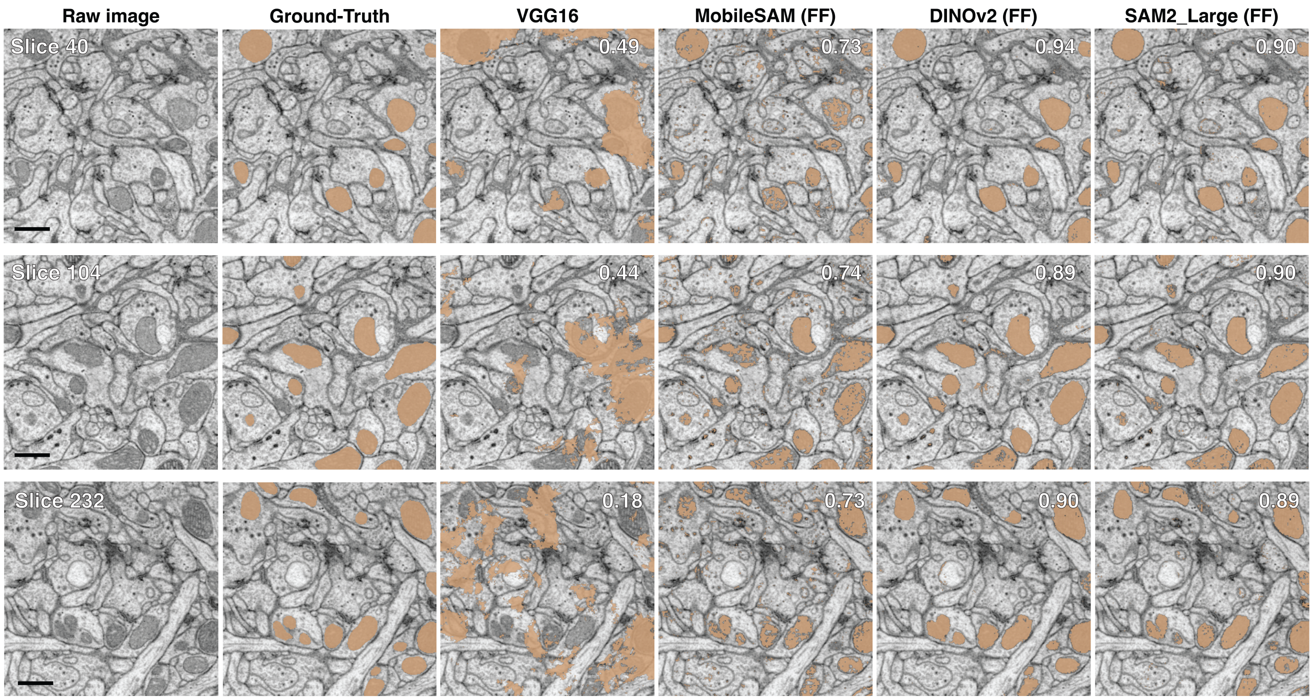

Supplementary Figure 10. Comparison of the different model performance on the Fly brain dataset. Dice score is reported in the corner of each relevant image.

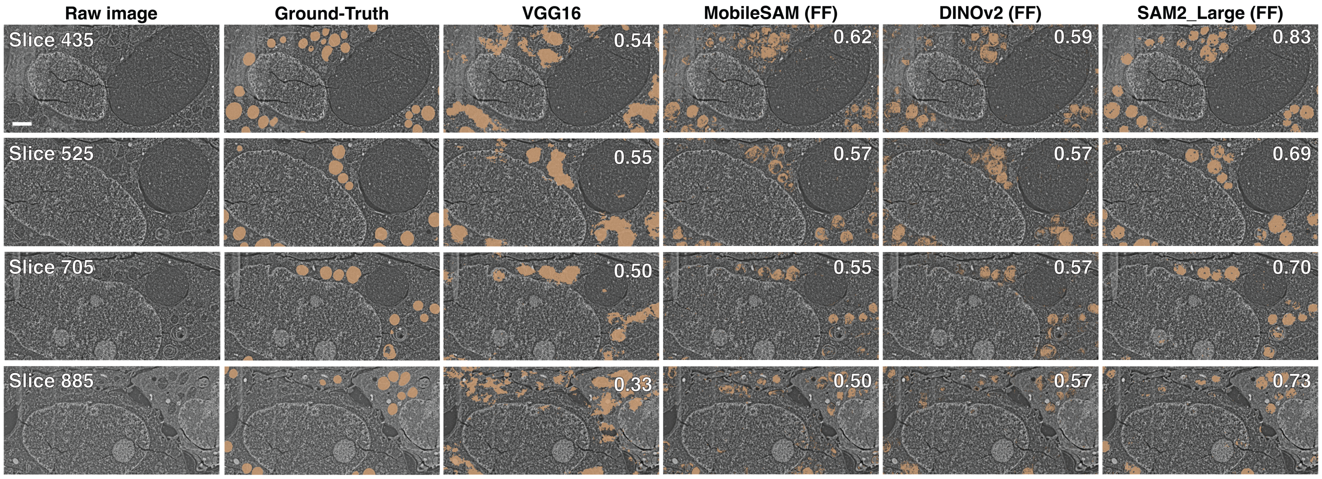

Supplementary Figure 11. Comparison of the different model performance on the Spheroid dataset. Dice score for the particular slice and method is reported in the corner of each relevant image. Scale bar 1 μm.

Dataset	Method	Dice	Precision	Recall	Boundary f1	Hausdorff
Fly brain	MobileSAM	0.69 ± 0.08	0.69 ± 0.10	0.70 ± 0.08	0.41 ± 0.07	9.79 ± 4.56
Fly brain	MobileSAM + PP	0.82 ± 0.09	0.92 ± 0.09	0.75 ± 0.09	0.76 ± 0.08	4.11 ± 3.79
Fly brain	DINOv2	0.89 ± 0.04	0.88 ± 0.04	0.91 ± 0.06	0.76 ± 0.07	1.49 ± 0.94
Fly brain	DINOv2 + PP	0.91 ± 0.07	0.89 ± 0.08	$\underline{0.93 \pm 0.06}$	0.88 ± 0.07	$\underline{1.34 \pm 2.31}$
Fly brain	FeatureForest	0.88 ± 0.05	0.91 ± 0.05	0.86 ± 0.06	0.70 ± 0.06	2.82 ± 2.04
Fly brain	FF + Post-Processing	$\underline{0.92 \pm 0.04}$	$\underline{0.97 \pm 0.03}$	0.87 ± 0.05	$\underline{0.92 \pm 0.05}$	1.49 ± 1.67
Spheroid	MobileSAM	0.57 ± 0.07	0.54 ± 0.10	0.61 ± 0.07	0.46 ± 0.07	29.85 ± 13.60
Spheroid	MobileSAM + PP	0.62 ± 0.09	0.57 ± 0.11	0.71 ± 0.08	0.56 ± 0.09	25.89 ± 15.64
Spheroid	DINOv2	0.56 ± 0.07	0.53 ± 0.08	0.61 ± 0.08	0.49 ± 0.06	28.61 ± 11.41
Spheroid	DINOv2 + PP	0.63 ± 0.08	0.54 ± 0.09	0.77 ± 0.08	0.53 ± 0.07	24.00 ± 12.08
Spheroid	FeatureForest	0.74 ± 0.06	0.76 ± 0.09	0.73 ± 0.06	0.71 ± 0.07	10.80 ± 7.74
Spheroid	FF + Post-Processing	$\underline{0.78 \pm 0.07}$	$\underline{0.78 \pm 0.10}$	$\underline{0.80 \pm 0.06}$	$\underline{0.79 \pm 0.07}$	$\underline{9.14 \pm 8.27}$

Supplementary Table 1. Post-processing performance with the FeatureForest models. The various metrics computed on the Fly brain and Spheroid datasets for the different models used for feature extraction (*MobileSAM*, *DINOv2*, *SAM2_Large*) and with SAM2 post-processing. The best performing method in each metrics is underlined, where Dice, precision, recall, and boundary F1 should be maximized, while Hausdorff distance should be minimized.

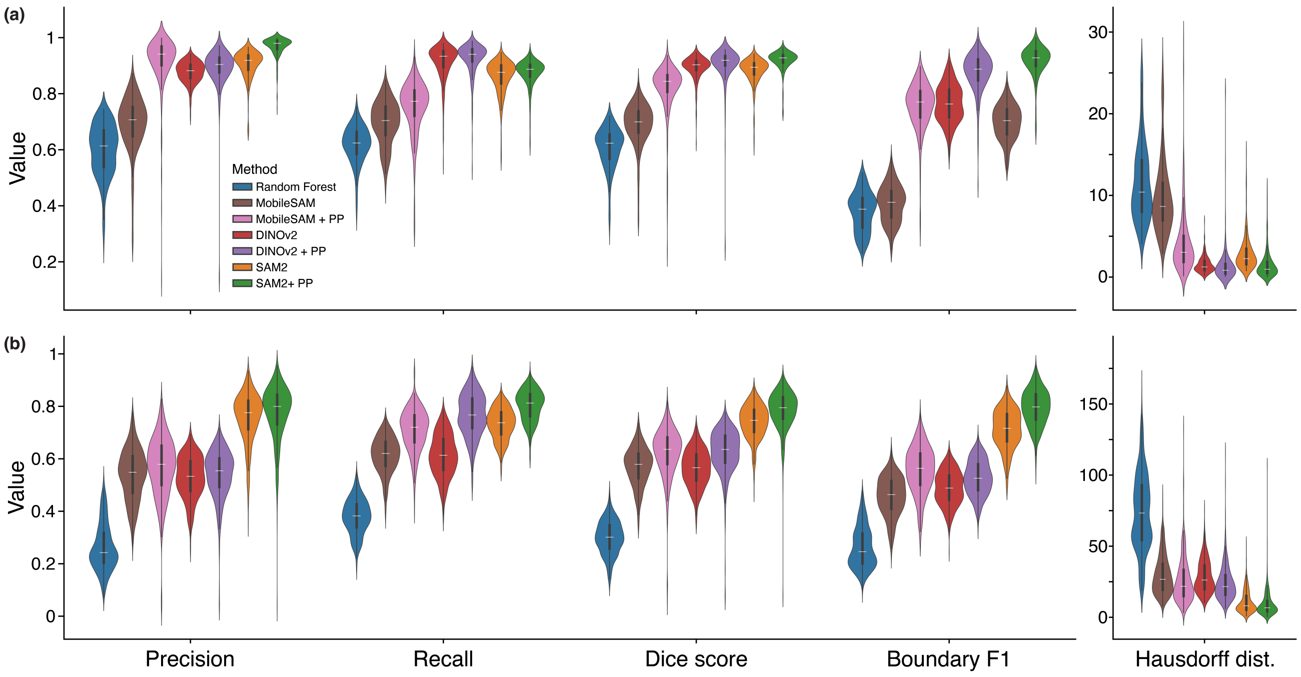

Supplementary Figure 12. Precision, recall, Dice score, boundary F1 and Hausdorff distance distributions for the different FeatureForest models with and without post-processing on the Fly brain and Spheroid datasets. (a) Metrics measured on the Fly brain dataset for the random forest classifier, and models available in FeatureForest: MobileSAM, DINOv2 and SAM2 (*SAM2_Large*) with and without SAM2 post-processing. Best performing methods reach higher values for precision, recall, Dice, and boundary F1, and lower values for Hausdorff distance. (b) Same for the Spheroid dataset.

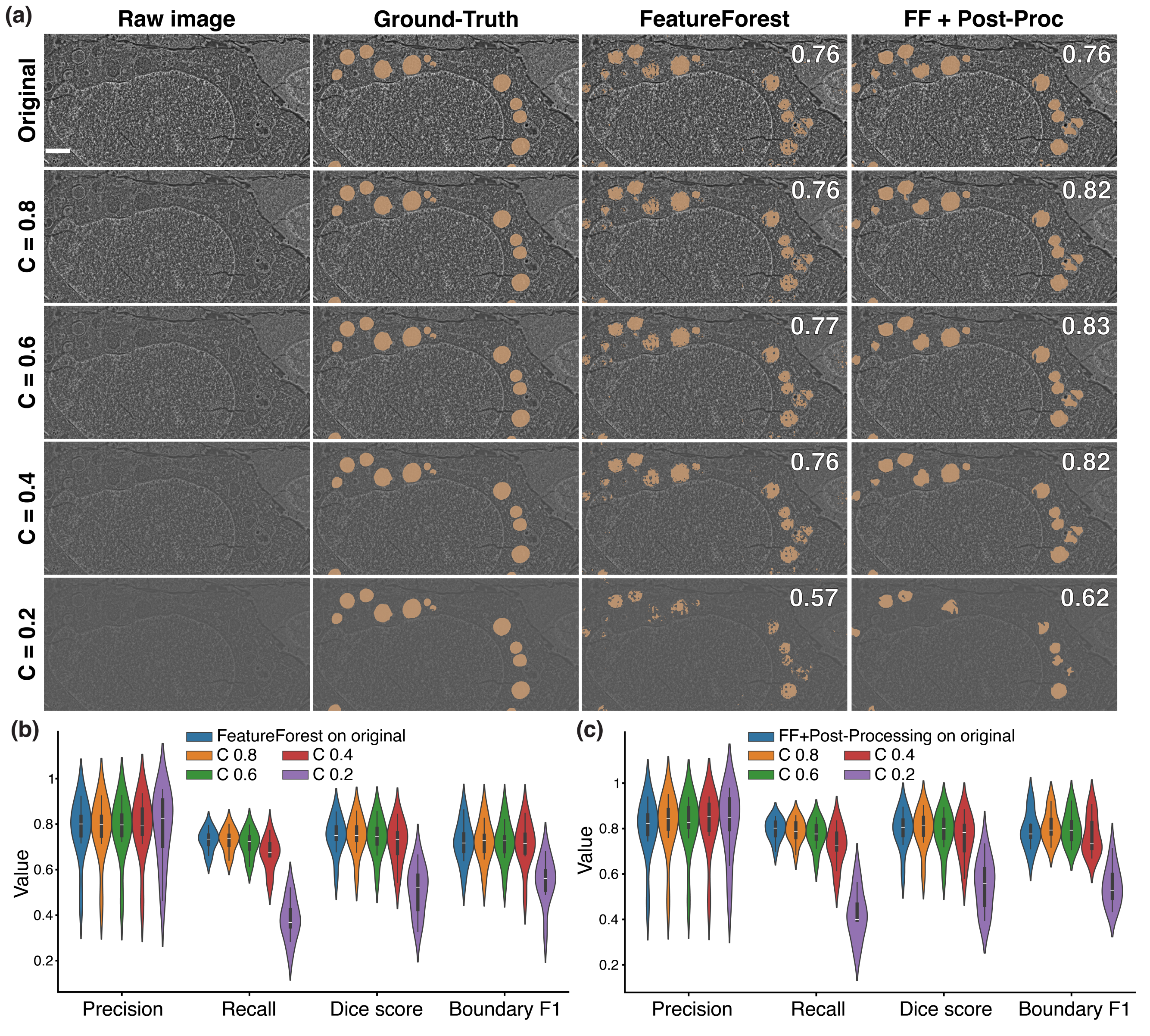

Supplementary Figure 13. FeatureForest on image with varying contrast. (a) Spheroid (slice 805) with varying contrast (*c*). Dice scores for the specific slice and methods are indicated in the top-right corner. Scale bar 1 µm. (b) Metrics computed over the test stack for FeatureForest (*SAM2_Large*) for each contrast level. (c) Same for FeatureForest with SAM2 post-processing.

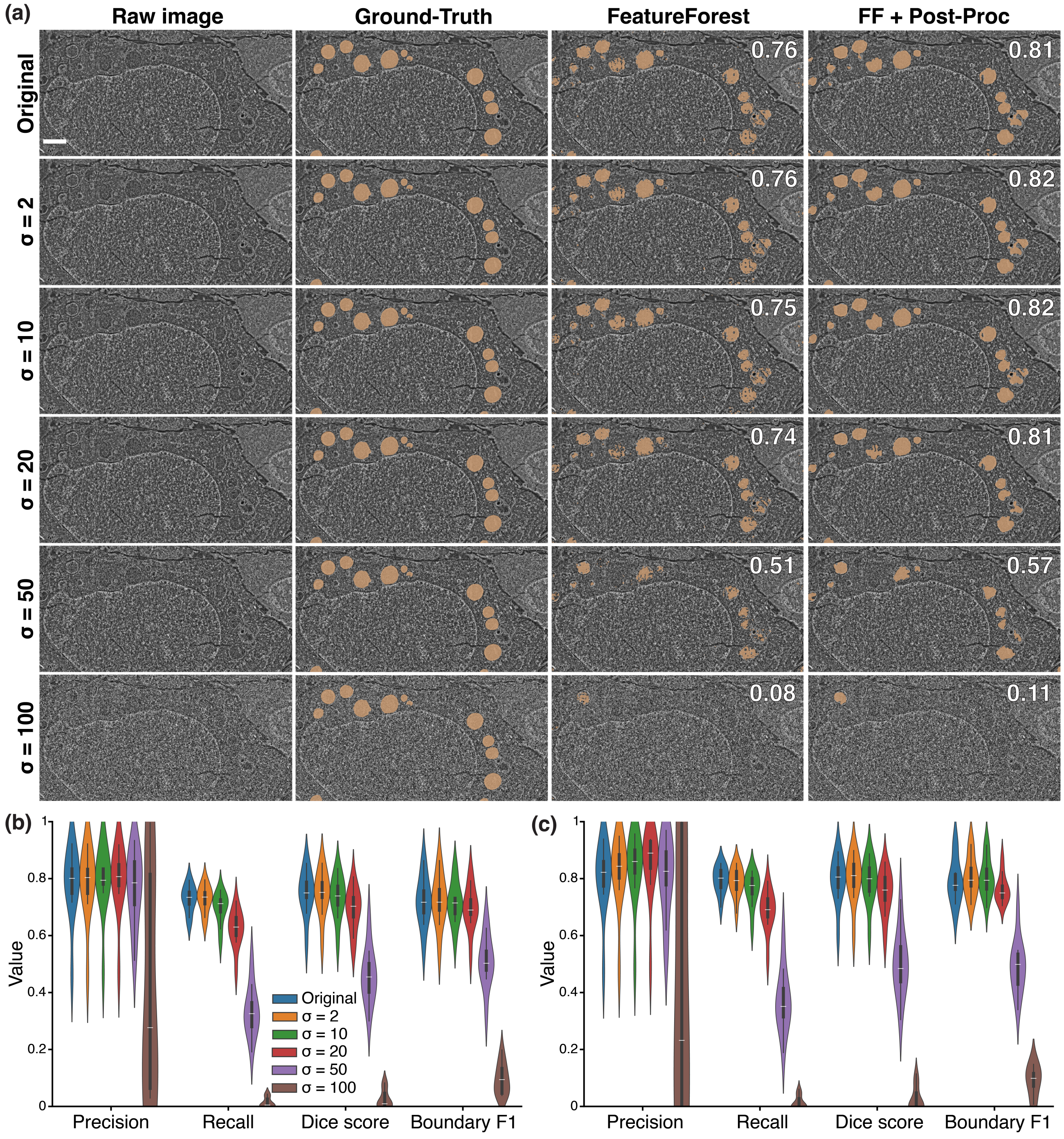

Supplementary Figure 14. FeatureForest on image with varying additive Gaussian noise. (a) Spheroid (slice 805) with varying additive Gaussian noise standard deviation (σ). Dice scores for the specific slice and methods are indicated in the top-right corner. Scale bar 1 µm. (b) Metrics computed over the test stack for FeatureForest (*SAM2_Large*) for each noise level. (c) Same for FeatureForest with SAM2 post-processing.

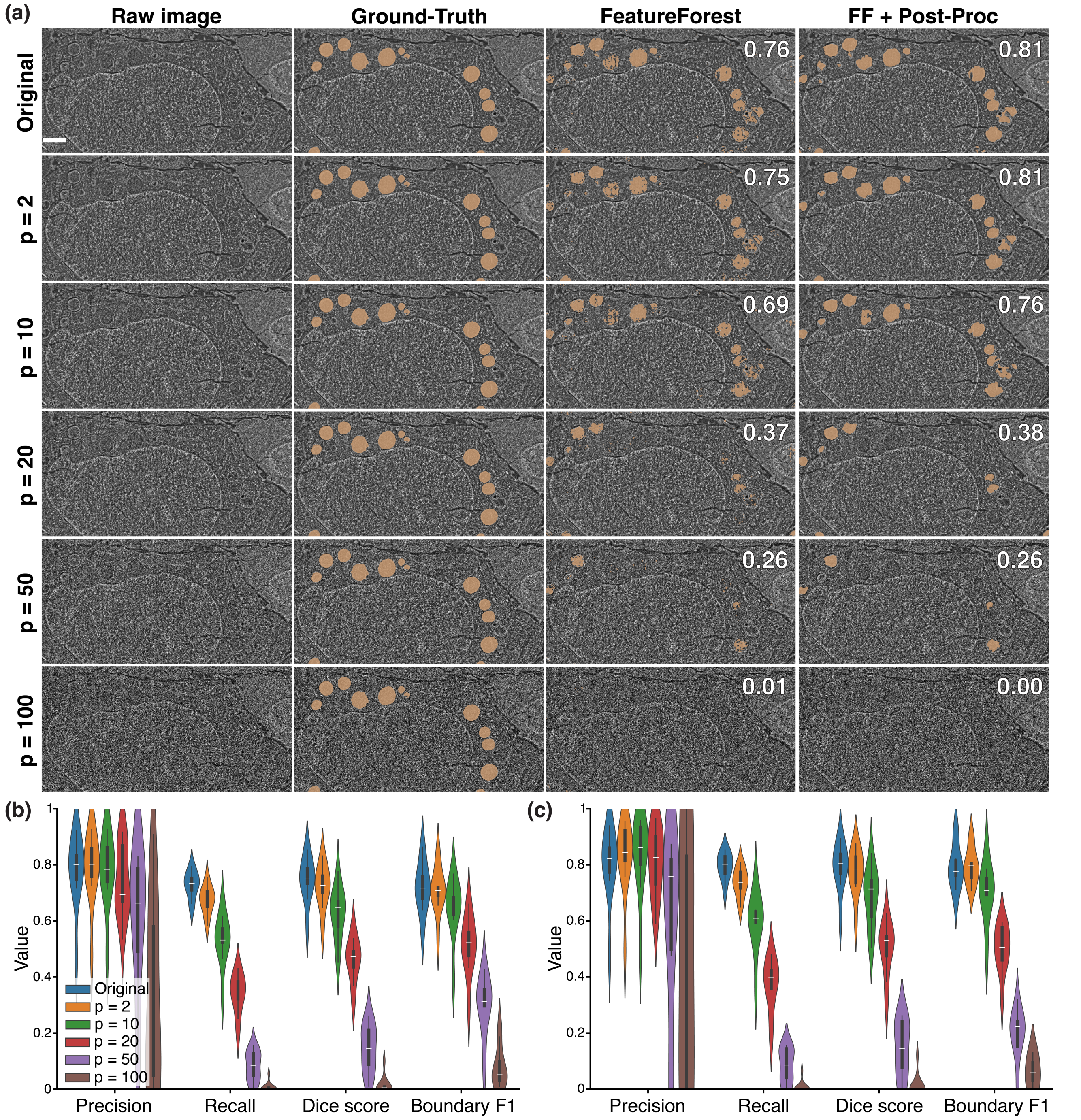

Supplementary Figure 15. FeatureForest on image with varying signal-to-noise ratio and Poisson noise. (a) Spheroid (slice 805) with varying intensity (p) corrupted by Poisson noise. Dice scores for the specific slice and methods are indicated in the top-right corner. Scale bar 1 µm. (b) Metrics computed over the test stack ($N = 10$) for FeatureForest (*SAM2_Large*) for each noise level. (c) Same for FeatureForest with SAM2 post-processing.

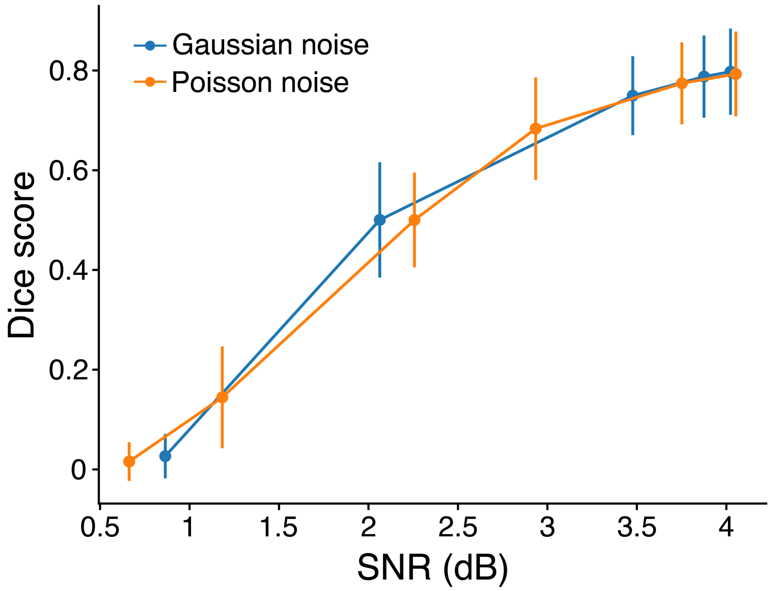

Supplementary Figure 16. Dice score of FeatureForest with post-processing against estimated signal-to-noise ratio.

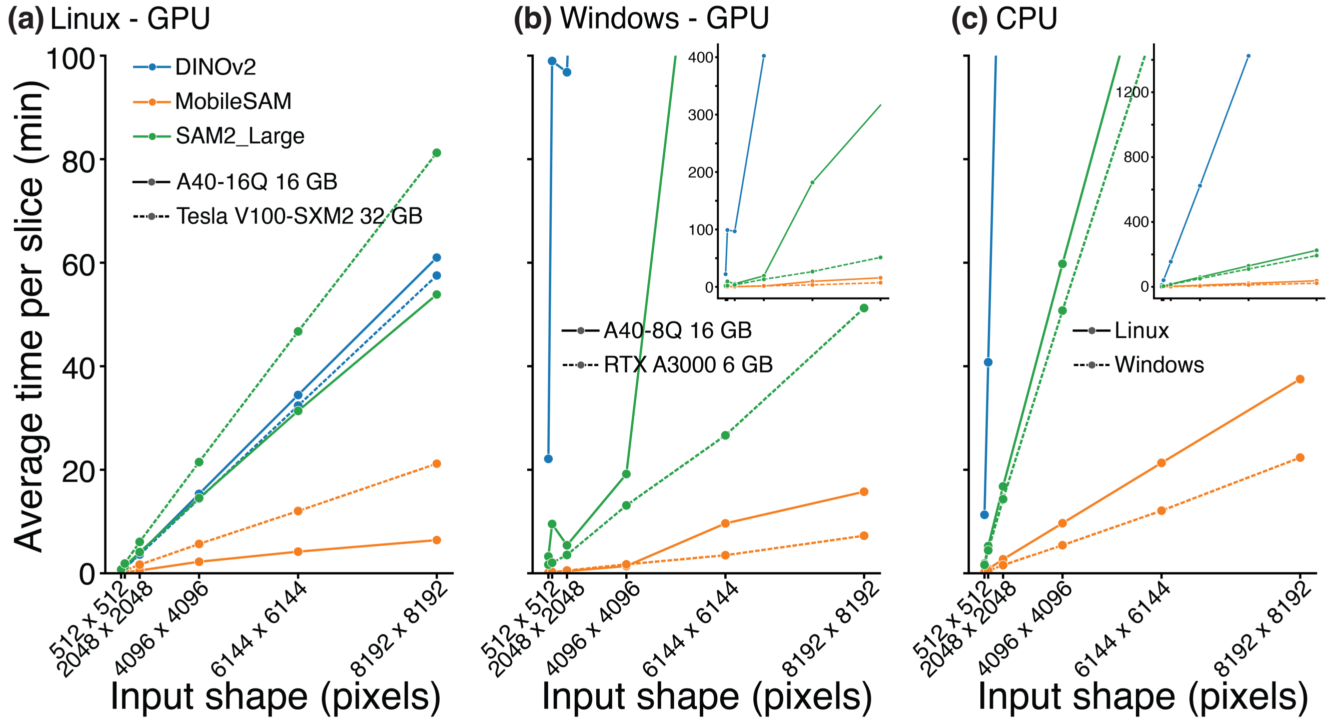

Supplementary Figure 17. Feature vectors extraction time for various input size, models and systems. (a) Average extraction time for a single slice of various size using the different FeatureForest models and for two GPUs on a Linux virtual machine (NVIDIA A40-16Q 16 GB) and an HPC GPU node (NVIDIA Tesla V100-SXM2 32 GB). (b) Same for two GPUs on a Windows virtual machine (NVIDIA A40-8Q 16 GB) and a laptop (NVIDIA RTX A3000 6 GB). Inset is the same graph without limiting the y-axis. DINOv2 did not run on the Windows virtual machine and was too slow to extract feature vectors for input larger than 4096×4096 in a decent amount of time. (c) Same for two different operating systems running the extraction on the CPU (Linux and Windows). Inset as in (b).

System	Model	Mean extraction time (s) 256×256	Standard deviation (s) 256×256
Linux 16 GB	MobileSAM	1.77	0.77
	DINOv2	3.58	0.36
	SAM2_Large	6.36	2.3
Linux 32 GB	MobileSAM	0.71	0.49
	DINOv2	4.07	1.11
	SAM2_Large	4.61	2.27
Windows 6 GB	MobileSAM	0.94	0.57
	DINOv2	221.92	150.30
	SAM2_Large	21.91	17.41
Windows 16 GB	MobileSAM	0.67	0.43
	SAM2_Large	7.48	8.81
Linux CPU	MobileSAM	2.83	0.97
	DINOv2	152.48	9.94
	SAM2_Large	17.37	5.79
Windows CPU	MobileSAM	1.61	0.52
	SAM2_Large	14.83	5.03

Supplementary Table 2. Feature vectors extraction time for various systems and models for a 256×256 **slice.** The systems are the following: virtual Red-Hat Linux machine with NVIDIA A40-16Q (Linux 16 GB GPU), Red-Hat HPC GPU node with NVIDIA Tesla V100-SXM2 (Linux 32 GB GPU), Windows enterprise laptop with RTX A3000 (Windows 6 GB GPU), Windows 10 enterprise virtual machine with A40-8Q (Windows 16 GB), Red-Hat Linux virtual machine (Linux CPU) and Windows enterprise laptop (Windows CPU).
